# Lipidomic Profiling in Synovial Tissue

**DOI:** 10.3389/fmed.2022.857135

**Published:** 2022-04-15

**Authors:** Roxana Coras, Jessica D. Murillo-Saich, Abha G. Singh, Arthur Kavanaugh, Monica Guma

**Affiliations:** ^1^Department of Medicine, School of Medicine, University of California, San Diego, San Diego, CA, United States; ^2^Department of Medicine, Autonomous University of Barcelona, Barcelona, Spain; ^3^San Diego VA Healthcare Service, San Diego, CA, United States

**Keywords:** lipidomics, synovitis, arthritis, synovial biopsies, inflammation

## Abstract

The analysis of synovial tissue offers the potential for the comprehensive characterization of cell types involved in arthritis pathogenesis. The studies performed to date in synovial tissue have made it possible to define synovial pathotypes, which relate to disease severity and response to treatment. Lipidomics is the branch of metabolomics that allows the quantification and identification of lipids in different biological samples. Studies in animal models of arthritis and in serum/plasma from patients with arthritis suggest the involvement of different types of lipids (glycerophospholipids, glycerolipids, sphingolipids, oxylipins, fatty acids) in the pathogenesis of arthritis. We reviewed studies that quantified lipids in different types of tissues and their relationship with inflammation. We propose that combining lipidomics with currently used “omics” techniques can improve the information obtained from the analysis of synovial tissue, for a better understanding of pathogenesis and the development of new therapeutic strategies.

## Introduction

### Synovial Tissue Pathology in Arthritis

The synovial tissue (synovium) is the main target of inflammation in rheumatoid arthritis (RA) and spondyloarthropathies (SpA) but it is also an important location of inflammation in other rheumatic diseases, such as osteoarthritis (OA). The synovium lines the diarthrodial joints, as well as tendons and bursae, and is comprised by a surface layer, the lining or intima, and the sublining or underlying tissue (1). Healthy synovium is characterized by a low cell content, where the lining contains 1–2 layers of cells, represented by macrophage- and fibroblast-like synoviocytes (FLS), while the sublining contains connective tissue with scattered blood vessels, fat cells, and FLS, with few lymphocytes and macrophages (1).

In pathological conditions, such as RA, SpA, or OA, the synovial membrane undergoes profound changes, with an increase in the number of infiltrating and proliferating cells as well neoformation of vessels (2). In RA, there is an increase of the thickness of the lining that becomes hyperplastic, both due to the proliferation of FLS and the recruitment of circulating macrophages. Circulating macrophages are also recruited to the sublining, which is hypercellular in RA and can also include FLS, T and B cells, as well as dendritic and mast cells (3).

Synovial pathology in SpA, and specifically in PsA, has been studied in comparison to RA, trying to identify features that differentiate both diseases. For the most part, PsA synovitis is similar to RA, including lining hyperplasia, neoangiogenesis and increased cellular infiltrates in the sublining layer. However, some differences are worth mentioning as they could have therapeutic implications (4). Macroscopically, the inflamed synovium of PsA exhibits bushy and tortuous vessels which is an expression of intense neovascularization, whereas the synovitis in RA predominantly shows straight and branched vessels (5). Although there seems to be no difference in the number of infiltrating cells, the PsA synovium contains a higher amount of mast cells, CD15+ neutrophils, and CD163+ macrophages (6). An important trait of PsA synovium is the high amount of IL17A loaded mast cells that inversely correlates with inflammation. These are cells with potential innate protective functions, and they are also present in other target tissues such as skin and gut. The regulation of the amount of IL17A in mast cells was proposed as potential therapeutic strategy in PsA (7).

The synovial pathology of OA includes not only synovitis but also fibrosis and contributes to both initiation and progression in OA. When present, synovitis is characterized by proliferation of FLS and recruitment of macrophages, resulting in hyperplasia of the synovial lining and cell infiltrating the sublining that include macrophages, T cells, and to a lesser extent, mast cells, B cells, plasma cells and endothelial cells (8). Macrophages are the most abundant immune cells in the synovial membrane of OA and are involved in both maintaining and resolving the inflammatory process. Moreover, inflammatory infiltrates coexist with fibrotic changes and angiogenesis in OA, which can be more prevalent in the late stages than in the early stages of the disease (8).

Synovial inflammation (or synovitis) causes joint pain and damage. Synovitis, when uncontrolled, is associated with disability, decreased quality of life, and increased morbidity and mortality (9, 10). Despite the existence of a wide range of therapeutic options for various rheumatic conditions, a number of patients do not respond well to current treatments. Some patients will experience persistent high disease activity (11); and even among responders, only a small percentage will reach remission (12). Furthermore, there are no disease modifying treatments available in OA, although it is the most prevalent type of arthritis. A better understanding of the pathogenesis of these diseases would offer opportunities for the identification of new targets and the development of new therapeutic interventions.

The use of synovial biopsies to determine new pathogenesis mechanisms has recently advanced the field in RA (13). Synovial tissue can be obtained by arthroscopy or with ultrasound guidance. The latter are minimally invasive procedures that can be performed by rheumatologists in the outpatient clinic, are safe and associated with a low risk of side effects (14). So far, most of the efforts have focused on the characterization of synovial tissue in RA and the information provided by the analysis of the tissue is highly relevant. Histological assessment combined with cell sequencing of the synovial membrane has led to a better characterization of the cell types involved in synovial inflammation and to the development of disease pathotypes in RA (15). As part of a large collaborative project, the Accelerating Medicines Partnership (AMP) consortium, functionally distinct FLS and macrophages among other cell populations were identified in the RA synovium (16). Of note DMARDs have an effect on FLS-macrophage crosstalk, paving the way for future applications in personalized medicine (17). Additionally, other studies have linked synovial pathotypes with disease severity and response to various targeted treatments (15, 18). These new techniques of sequencing for a better characterization of the synovial cells may not yet be able to capture certain functional features of the tissue and cells, such as metabolic activity. The addition of lipidomics to the other “omic” techniques in the study of synovial tissue will make possible to further deepen the characterization and understanding of the pathogenesis of the disease, identifying different lipid metabolic pathways specifically altered in the different types of arthritis. Moreover, the identification of different metabolic profiles has the potential to discover new therapeutic targets and predictors of disease progression and response to treatment.

### Lipid Metabolism in Inflammation

Classically, lipids have been described as the main components of cell membranes, and also used as fuel and energy storage. In the recent years, studies have shown that lipids are bioactive molecules and function as signaling molecules, participating in the regulation of several cell processes such as cell death, proliferation, and inflammation (19).

**Figure 1:**
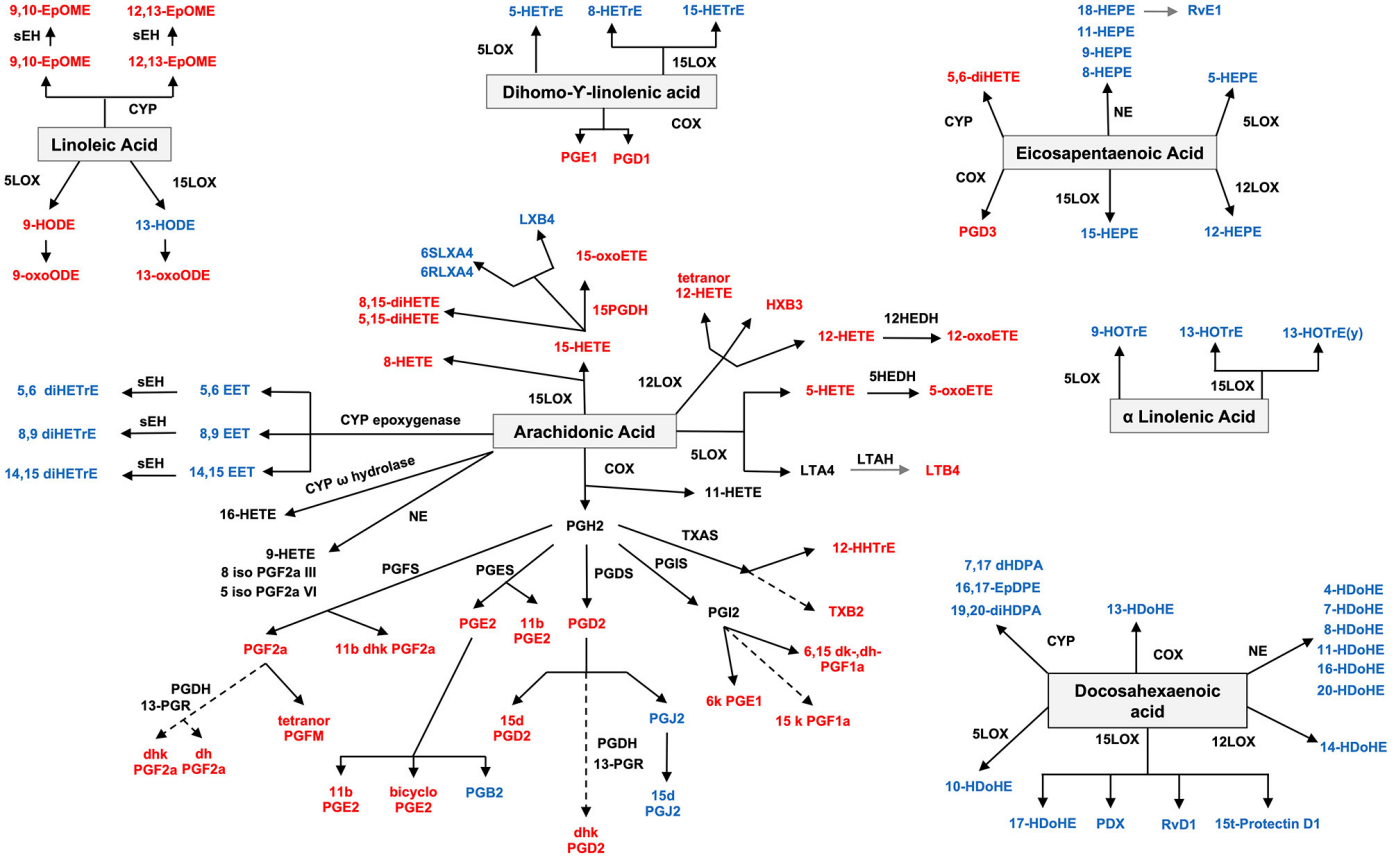
Oxylipin synthesis. Pro-inflammatory oxylipins are marked in red, while anti-inflammatory ones are marked in blue. LOX, lipoxygenase; CYP, cytochrome; NE, non-enzymatic; PGDS, prostaglandin D synthase; PGFS, prostaglandin F synthase; PGES, prostaglandin E synthase; PGIS, prostaglandin I synthase; HEDH, Hydroxyeicosanoid dehydrogenase; LTAH, Leukotriene A4 hydrolase; PGDH, hydroxy prostaglandin dehydrogenase; TXAS, thromboxane A synthase; PGR, 15-ketoprostaglandinΔ13 reductase; sHE, soluble epoxide hydrolase.

**Figure 2:**
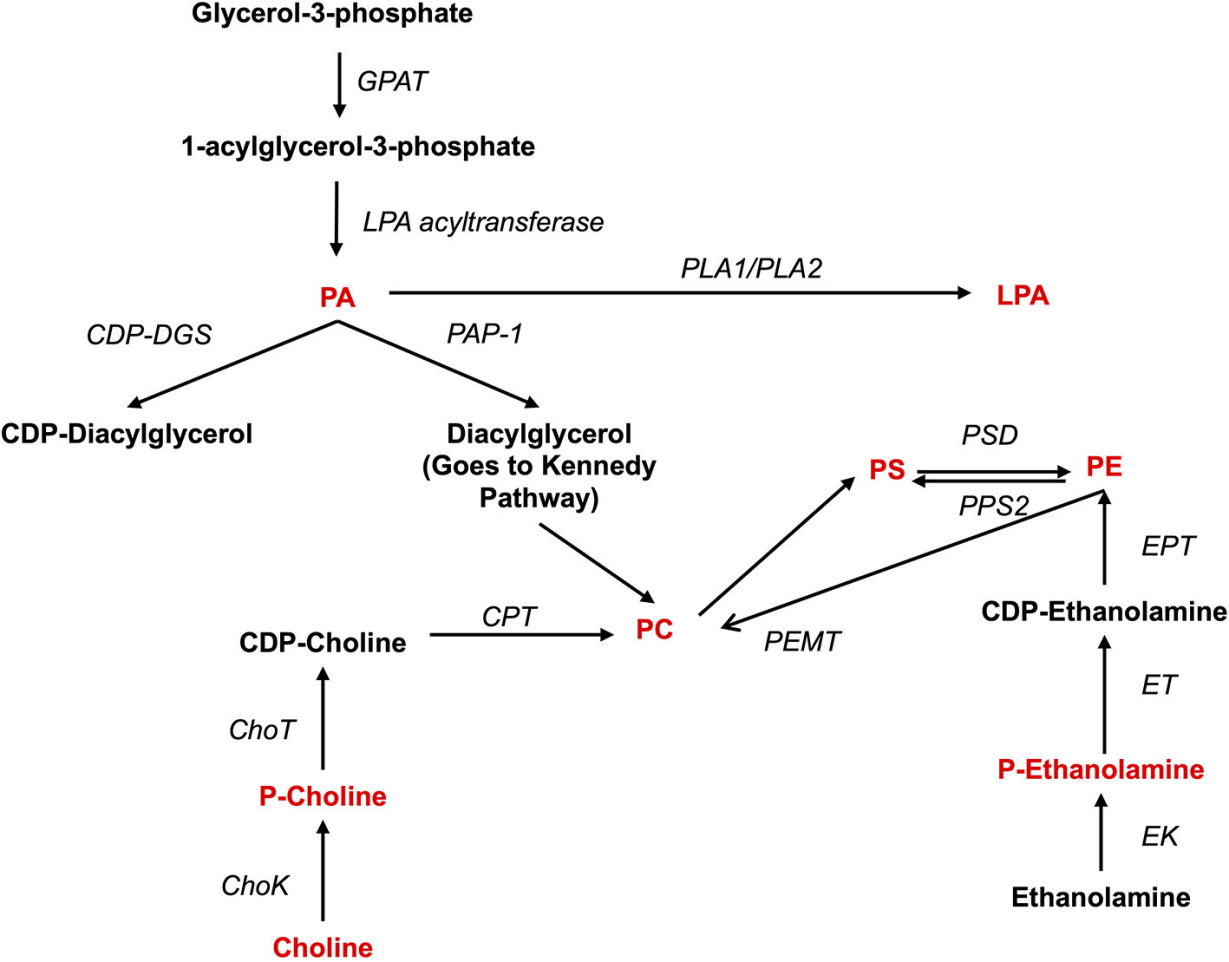
Synthesis of phospholipids. Pro-inflammatory lipids are marked in red, while anti-inflammatory ones are marked in blue. ChoK, Choline Kinase; CDP-DGS, Cytidine diphosphate diacylglycerol Synthase; CPT, Carnitine Palmitoyltransferase; GPAT, glycerol-3-phosphate acyltransferase; ChoT, CholineTRansferasa; EK, Ethanolamine kinase; ET, Ethanolamine transferase; EPT, Ethanolalmine phosphotransferase; LPA acetyltransferase, lyso-phosphatidic acid acyltransferase; PA, Phosphatidic acid; PAP-1, Phosphatidate phosphatase-1; PC, Phosphatidylcholine; P-Choline, Phosphocholine PE, Phosphatidylethanolamine; P-Ethanolamine, Phosphoethanolamine; PLA1, Phospholipase A1; PLA2, Phospholipase A2; PEMT, PE methyltransferase; PS, Phosphatidylserine; PPS2, PS synthase 2; PSD, PS-decarboxylase.

**Figure 3:**
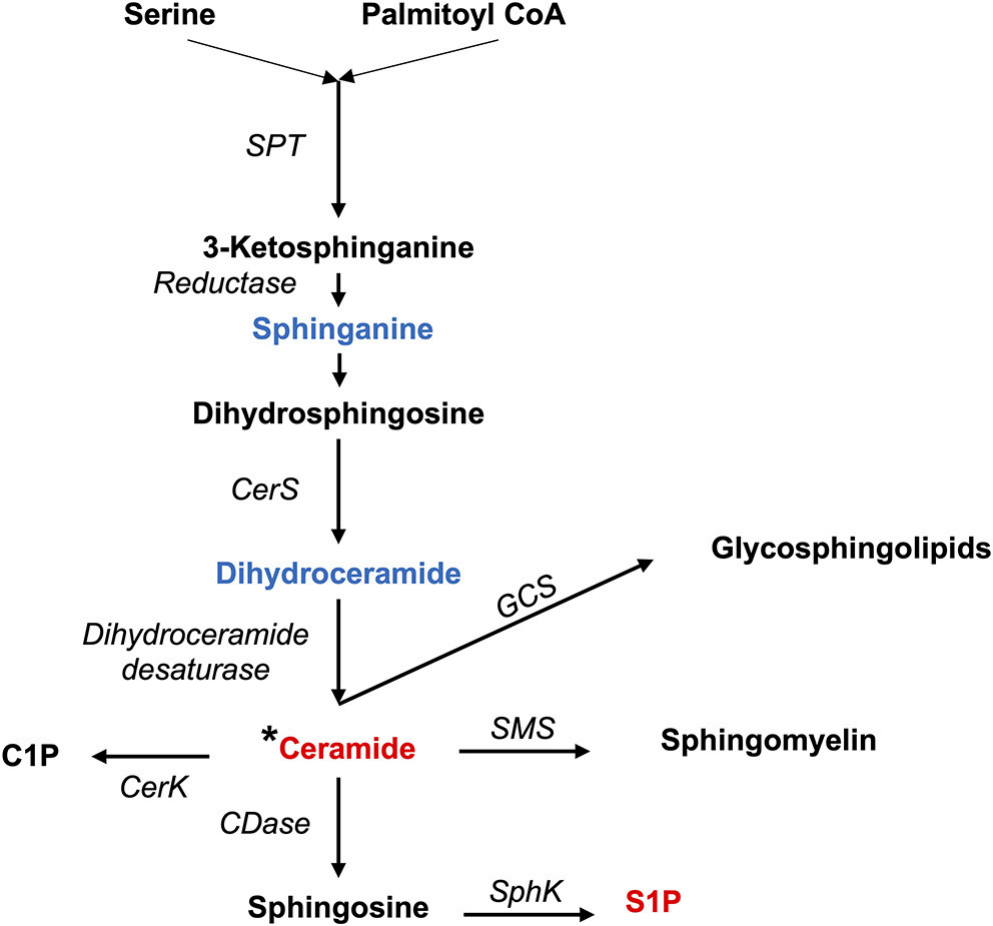
Synthesis of sphingolipids. Pro-inflammatory lipids are marked in red, while anti-inflammatory ones are marked in blue.C1P, Ceramide 1-phosphate; CDase, Ceramidase; CerK, Ceramide Kinase; CerS, Ceramide Synthases 1–6; GCS, Glucosylceramide Synthase; SMS, Sphingomyelin synthase; SPT, Serine Palmitoyltransferase; S1P, Sphingosine 1-phosphate; SphK, Sphingosine kinase; *Different types of ceramides can act as either proinflammatory or anti-inflammatory.

Both pro- and anti-inflammatory lipids are involved in the pathogenesis of arthritis. The type of lipids better characterized in arthritis are the oxylipins (Figure 1). The pro-inflammatory oxylipins derived from omega 6 polyunsaturated fatty acids (PUFA) such as arachidonic acid (AA) are responsible for some of the clinical symptoms of arthritis, such as pain, swelling and stiffness. Anti-inflammatory and specialized pro-resolving mediators (SPM) are synthesized from omega 3 PUFA such as DHA or EPA and are critical for the resolution of inflammation and return to homeostasis. Other lipid classes expanded in this review, such as glycerophospholipids and sphingolipids have been less studied in the context of inflammatory arthritis. These are components of cellular and organelle membranes that regulate functions such as membrane shaping, cell trafficking, cell growth and death, inflammatory cascades, and leukocyte adhesion (20). A few glycerophospholipids, including phosphatidylcholine (PC), phosphatidylserine (PS), phosphatidylethanolamine (PE), and phosphatidic acid (PA) have proinflammatory properties (Figure 2). As for the sphingolipids (Figure 3), some ceramides [Cer(d18:1/24:2) and Cer(d18:1/24:0)] are increased in inflammatory processes, while other ceramides, sphinganine and dihydroceramides are associated with decreased inflammation (20). Importantly, both FLS and immune cells, either resident or recruited in the synovial tissue can secrete bioactive lipid mediators.

Several drugs employed in the treatment of arthritis can target different pathways belonging to the lipid metabolism [reviewed elsewhere (21)], further supporting the involvement of lipids in arthritis. Non-steroid anti-inflammatory drugs (NSAIDs), commonly used to treat inflammatory arthritis, inhibit the synthesis of prostaglandins and leukotrienes, by acting on the cyclooxygenase (COX) enzymes involved in their synthesis (Figure 1). The mechanism of action of glucocorticoids also involves effects on the lipid metabolism: they promote fatty acid synthase and acetyl-CoA carboxylase activation and inhibition of fatty acid β-oxidation by blocking acyl-CoA dehydrogenase activity. Other treatments, including hydroxychloroquine, methotrexate, and biological therapies have beneficial effects of on the lipid profile (21).

## Types of Lipids and Methods Employed in Lipidomics

Lipidomics is a branch of metabolomics which involves “the full characterization of lipid molecular species and their biological roles with respect to the expression of proteins involved in lipid metabolism and function, including gene regulation” (22). Lipids are complex molecules and can be classified in several chemical classes (Table 1), as well as extracted and identified using several methods (Table 2).

**Table 1:** Lipid classification according to LIPID MAPS.

**Lipid type**	**Chemical group**	**Structure**	**Subclass**
Fatty acid (FA)	Carboxylic acid (COOH) group bonded to a saturated carbon backbone.	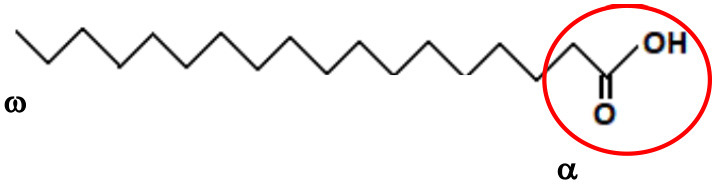	FA (Capric acid, Lauric acid, Oleic acid, Linoleic acid) and FA conjugates: Fatty esters, Fatty alcohols, Fatty amides, Oxylipins
Glycerolipids (GL)	Glycerol-backbone. Long-chain acyl and alkyl groups, and a collection of polar alcohols	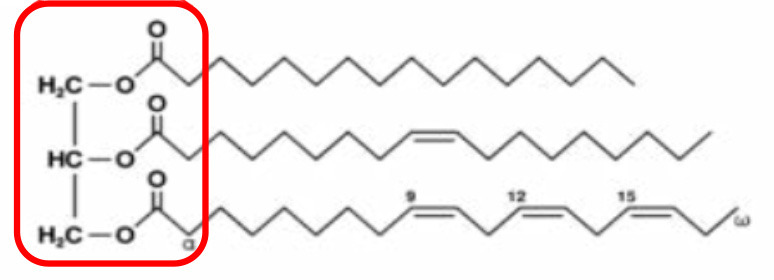	Monoacylglycerol (1 FA) Diacylglycerol (2 FA) Triacylglycerol (3FA)
Glycerophospholipids (GLP)	Glycerol-backbone. Terminal ester group (X) are ethanolamine, choline, serine or inositol. It has a phosphate headgroup and R indicates FA	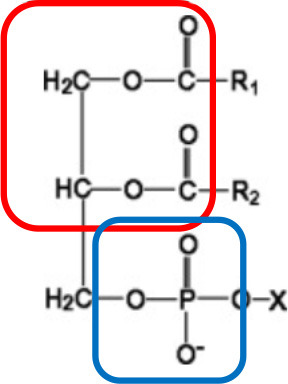	Phosphatidic acid Phosphatidylcholine Phosphatidylserine Phosphatidylethanolamine Phosphatidylinositol Phosphatidylglycerol Cardiolipins
Sphingolipids (SP)	Backbone of sphingosine bases and **set of aliphatic amino alcohol**. R indicates FA	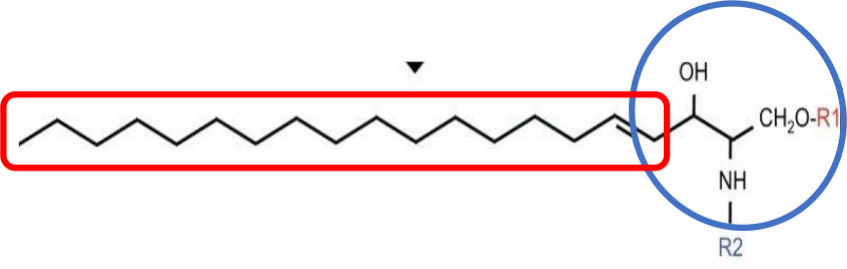	Ceramide Sphingosine Sphingomyelin Glycosphingolipid Sphingosine 1-P

**Table 2:** Examples of extraction methods and Chromatography-Mass Spectrometry.

**Type of sample**	**Method of extraction**	**Solvent**	**Technique**	**Type of lipid**	**References**
Plasma	SPE using Enhance Matrix Removal—EMR Lipid	ACN/MeOH (95:5, v/v)	LC-MS	FAs, PLs, SPs.	(23)
Plasma and serum	LLE	CHCl_3_/MeOH (1:1, *v/v*)	LC-MS	TG, PLs, SPs	(24)
Plasma	SPE (oxylipins) LLE (rest of lipids)	Methanolic HCl/isooctane (1:3, v/v).	GC-MS, LC-MS	Oxylipins, GLs, GPLs, SPs	(25)
Brain sample	LLE	CHCl_3_/MeOH (1:1, *v/v*)	LC: MS	SPs	(26)
Macrophages	LLE	C8H19N/ PFB-Br (1:1, in ACN)	GC-MS	FAs	(27)
Synovial tissue	LLE	CHCl_3_/MeOH (2:1, *v/v*)	MALDI-MSI	FAs, SPs, GPs	(28)

*SPs, sphingolipids; GLs, glycerolipids; GPLs, glycerophospholipids; FA, fatty acids; TG, triacylglycerol; PL, phospholipids; LC-MS, liquid chromatography-mass spectrometry; GC-MS, gas chromatography-mass spectrometry; LLE, liquid-liquid extraction; SPE, solid phase extraction; CHCl_3_/MeOH, chloroform/methanol; C8H19N, diisopropylethylamine; PFB-Br, pentafluorobenzyl bromide; ACN, acenotrile*.

### Classification of Lipids

Lipids are chemical compounds with different bioactive functions. LIPID MAPS (https://www.lipidmaps.org/), an online resource for lipidomics, has classified lipids into 8 groups according to the presence of ketoacyl and isoprene groups. Based on this classification system, lipids have been divided into eight categories: fatty acyls, glycerolipids (GLs), glycerophospholipids (GPLs), sphingolipids (SPs), saccharolipids, polyketides (derived from condensation of ketoacyl subunits), sterol lipids, and prenol lipids (derived from condensation of isoprene subunits) (29) (Table 1). Fatty acids (FAs), included in the fatty acyl category, are the main component of most of these lipids. The general structure of a FA consists of a straight chain of an even number of carbon atoms (also named acyl chain), with hydrogen atoms along the length of the chain at one end of the chain and a carboxyl group (—COOH) at the other end. Depending on the number of double bonds, they are classified as saturated (without double bonds in the acyl chain), monounsaturated (MUFA, with one double bond), or polyunsaturated (PUFA, with more than 2 double bonds). Additionally, they can be classified based on the number of carbon atoms, as short-chain FAs (SCFAs), with up to 6 carbons, medium-chain FAs (MCFAs), with 6–12 carbons, long-chain FAs (LCFAs), with more than 12 carbons, or a recently discovered subgroup of the latter group which has been defined as very long-chain fatty acids (VLCFAs), with more than 22 carbons. The length of the acyl chain and the degree of its saturation determine the various functions of FAs, such as the rigidity of the plasma membrane and the biological effects in humans. The degree of unsaturation determines the susceptibility of the unsaturated FAs to oxidation, which makes the membrane resistant to damage or penetration by drugs (30).

**Figure 4:**
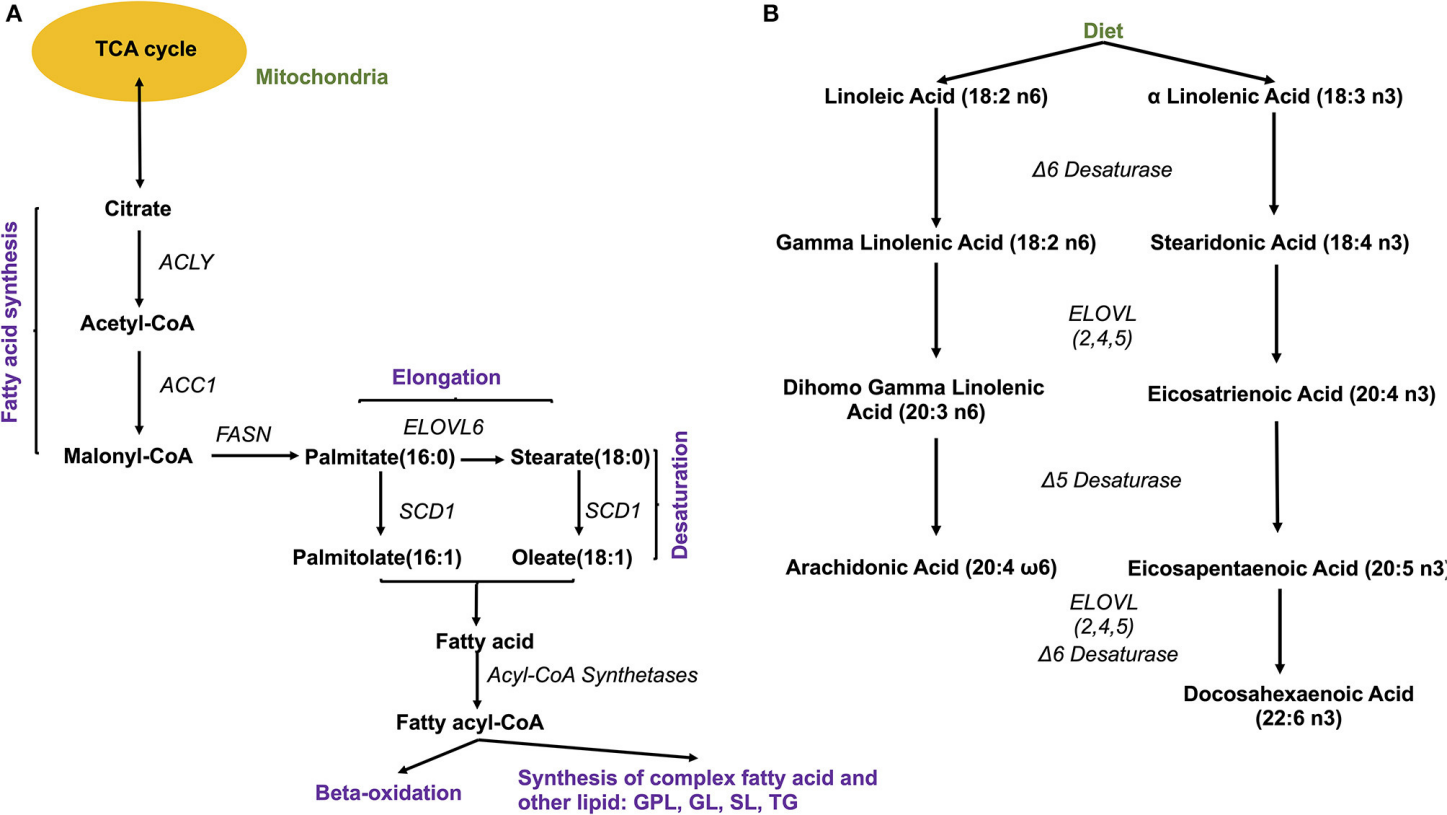
Fatty acid synthesis. **(A)** *De novo* synthesis of endogenous fatty acids; ACYL, ATP citrate lyase; ACC1, Acetyl-coenzyme A carboxylase 1; FASN, fatty acid synthase; SCD1, stearoyl-CoA desaturase 1; ELOVL, elongase 6; **(B)** Synthesis of PUFA from essential fatty acids. ELOVL 2,4,5, elongases 2,4,5.

The source of FAs can be both endo- and exogenous (Figure 4). Endogenously, *de novo* synthesis of FA from acetyl-coenzyme A (acetyl-CoA) is catalyzed by fatty acid synthase (FASN) yielding palmitate (16:0), which can then be either desaturated to palmitoleate (16:1) by stearoyl-CoA desaturase 1 (SCD, a delta 9 desaturase), or elongated by an elongase (ELOVL6) to stearate (18:0). Stearate, a saturated FA, is then converted into oleate (MUFA) by SCD, and its chains are elongated by elongases (ELOVL). There are 2 groups of elongases: ELOVLs 1, 3 and 6, involved in the elongation of saturated FAs and MUFAs, and ELOVLs 2, 4 and 5, which are responsible for the elongation of PUFAs (31).

The other source of FA is exogenous, since some FAs cannot be synthesized by human cells due to the lack of the enzymatic system that introduces double bonds at position omega (n)-6 (carbon 6 from the omega end) or lower. Both 18:3 n-3 FAs (alpha linolenic acid, α-LNA), found in some plant oils (flaxseed, rapeseed, canola), walnuts and leafy greens, and 18:2 n-6 FAs (linoleic acid, LA), contained in meat, poultry, cereal products, and oil, are essential FAs and must be provided with the diet. Once ingested, they serve as precursor for other n-3 (eicosapentaenoic acid, EPA, 20:5 n-3, docosapentaenoic acid, DHA, 22:6 n-3) or n-6 (arachidonic acid, AA, 20:4 n-6) PUFAs, with the intervention of several elongases (ELOVL) and FA desaturases (FADS) such as FADS1, a delta 5 desaturase—rate-limiting enzyme that introduces a double-bond at the 5th carbon of the n-3 and n-6 PUFA chain, and FADS2, a delta 6 desaturase- rate limiting enzyme that introduces a double-bond at the 6th carbon in the FA chain) (Figure 4).

FAs then need to be activated, as FA-CoA, to be able to perform biological roles (32). The activated FAs are either transported to mitochondria for oxidation and energy generation or serve as substrates for the synthesis of other categories of lipids, such as GLs, GPLs, and SPs (Table 1). These categories of lipids have a headgroup that binds to a backbone, which have a high structural variability and are responsible for a large range of functions, including membrane curvature, cell signaling and substrate transport (33). The essential FAs, AA, EPA, and DHA are commonly used for the synthesis of classical [prostaglandins (PG), leukotrienes (LT), thromboxanes (TX) and lipoxins (LP)] and non-classical (endocannabinoids (eCBs), neuroprotectins and resolvins) oxylipins (33).

### Extraction Methods and Chromatography-Mass Spectrometry

Lipids are embedded in complex matrices (blood, tissue, urine), therefore, prior to analysis, an isolation/fractionation step is necessary to remove non-lipid molecules such as proteins, sugars, and other small molecules. Two methods are widely used for the separation of lipids from biological samples. The first is called liquid-liquid extraction (LLE) and allows for the instant partitioning of the lipids. It uses a chloroform/methanol solvent with or without incorporated water as the extraction matrix. The high efficiency of this extraction method is due to the capability to penetrate through the cell membrane, the higher polarity, and the stronger interaction with the hydrogen bond.

The second method, solid-phase extraction (SPE), does not require partitioning of lipids in a solvent/water mixture, but uses stationary materials, such as bonded silica gel with –CN, –NH2, or diol groups, in combination with different elution solvents for lipid separation. The application of solvents with increasing polarity allows efficient isolation of phosphatidylcholine (PC), non-esterified FAs, cholesterol esters and triacylglycerols (TG). This method can also be employed for the fractionation of lipid subclasses, including ceramides, GPLs, sphingomyelins (SMs) and phosphorylated sphingoid bases. Compared to LLE, SPE shows an improved recovery and selectivity for phospholipids (PLs), including GPLs and phosphosphingolipids. SPE has the advantages of simplicity in operation, reduced solvent cost, and easy automation over liquid-liquid extraction, leading to its increasing popularity, especially in targeted lipidomics while liquid-liquid extraction tends to be used for non-targeted lipid profiling (13).

After extraction, lipids from biological samples are dissolved in a solvent and undergo separation, using either gas chromatography (GC) or liquid chromatography (LC) depending on physical state of mobile phase used, followed by detection of spectra using mass spectrometry (MS). The gases more commonly used in GC are helium, nitrogen, argon and hydrogen, while LC uses solvents with high polarity such as water, methanol and acetonitrile. LC is the chromatographic technique most commonly used for lipids. However, due to the chemical diversity and physicochemical characteristics of each lipid variation, the selection of the methods, solvents and the chromatographic method need to be performed with caution (34). Table 2 shows a few examples of extraction, separation, and detection methods used to quantify lipids from different types of biological samples, along with the types of lipids identified by these methods.

Circulating levels of lipids can be influenced by a variety of factors that include diet, gut microbiome and absorption, age, sex, comorbidities, physical exercise, and drugs. This certainly makes the interpretation of the data more difficult (35). Yet, no data is available on the effect of all these factors on the lipid composition of the synovial tissue, emphasizing the need to study lipidomics in this tissue.

## Evidence of Lipid Alterations in Arthritis

There is at present a relative paucity of lipidomics studies performed on synovial tissue to assess the lipid classes and subclasses present in this tissue. Isolated cells from RA patients who underwent synovectomy were described to produce PGE2, which was suppressed by indomethacin and dexamethasone, suggesting an involvement of PGE2 in the pathogenesis of RA (36). However, lipidomics has not been largely used in the study of synovial tissue. An exception is several studies by Rocha et al., who found that osteoarthritis (OA) synovium presents elevated levels of PC, FAs and lysophosphatidic acids, and lower levels of lysophosphatidylcholines (LysoPC) compared to control tissues. Moreover, the spatial distribution of specific GPLs was also correlated with hypertrophic, inflamed and vascularized synovial areas. Compared to other inflammatory arthritis, the OA tissue showed lower amounts of phosphatidylethanolamine (PE)-based plasmalogens (28). The second study of the same group compared lipidomics profiles in rheumatoid arthritis (RA, *n* = 6), psoriatic arthritis (PsA, *n* = 12), and control donors (*n* = 10). Amongst the 35 lipid species that were significantly different between the groups, PC and PE, such as PE 34:1 and PE 36:1, were higher in RA and PsA compared to controls. Additionally, the spatial distribution of the mentioned PE species was associated with areas of the sublining layer with increased vascularity and inflammatory cell infiltrates, and their levels were also increased in synovial fluid (SF) from PsA patients compared to RA (37).

### Oxylipins

Studies on gene expression in synovial tissue and in animal models, though, suggest a role for lipid pathways. The most studied lipids in the pathogenesis of RA are the oxylipins, namely PG, LT and TX, which are derived from AA through COX and LOX enzymes and are considered to have a pro-inflammatory role (Figure 1). Cytosolic phospholipase A2, the enzyme that releases FAs from membranes, is overexpressed in SF from RA patients (38) and is induced by IL-1b in FLS (39). The expression of COX2 (inducible) but not COX1 (constitutive) is increased in synovial explants from RA patients (40, 41), as well as in synovium from RA, ankylosing spondylitis (AS), and PsA compared to OA, by immunohistochemistry (IHC) and mRNA expression (42). Downstream enzymes of COX2, such as prostaglandin E synthase (PGES), specifically the inducible microsomal isoform 1 (mPGES1), involved in PGE2 synthesis, is also overexpressed in both synovial tissue and cartilage and contributes to chronic inflammation (43). Specifically, RA is characterized by an upregulation of the COX2-mPGEs1-PGE2 axis. Pro-inflammatory cytokines (IL-1b, TNF, and lipopolysaccharide) induce the expression of COX2 and mPGES1 and secretion of PGE2 in RA FLS and mononuclear cells in RA SF (44, 45). In addition, PGI2, PGF2a, and 8-iso-prostaglandin F2α were elevated in SF and urine of RA patients suggesting pro-inflammatory effects (46–48) [reviewed (49)]. In animal models, the genetic deletion of mPGES1 in the collagen induced arthritis (CIA) model was associated with decreased severity of arthritis (50). PGE2 has a role not only in initiating and maintaining inflammation, but also in pain in RA. Other PG related oxylipins, such as PGD2 and 15-deoxy-D12,14-prostaglandin J2 (15d-PGJ2) decrease inflammation in animal models of arthritis (51).

The COX2-mPGES1-PGE2 axis is also upregulated in OA, although less data is available. PGE2 is increased in both synovial tissue and SF from OA patients (52, 53). Similar to RA, proinflammatory cytokines (IL-1β, TNF, or IL-17) induced expression of mPGES-1, and enhanced PGE2 production in OA chondrocytes and synovial fibroblasts (54).

The effect of the antirheumatic drugs on the synovial expression of COX2 and mPGES1 is somewhat surprising. In RA patients, intraarticular glucocorticoids are associated not only with clinical improvement, but also decreased synovial expression of COX1 and 2 and mPGES1, as well as decreased PGE2 production (44, 55). However, treatment with methotrexate (55) and TNF inhibitors was followed by no change in the expression of these enzymes not the amount of PGE2 in the synovial tissue (44). These are important observations as they might explain why a percentage of patients does not respond to these treatments. Therefore, adding lipidomics to the study of synovial tissue, might uncover active pathways despite treatment pointing at new potential therapeutic targets.

Other enzymes, 5-, 12- and 15 lipoxygenases (LOX), are also expressed in human synovial tissue and synoviocytes (56, 57). 5-LOX mRNA was detected in RA synovial tissue, specifically in macrophages in the lining (57, 58). Leukotriene B4 (LTB4, AA derived oxylipin *via* 5LOX) and its receptor BLT1 are critical for the development of arthritis in the K/BxN mouse model (59, 60). Inhibition of 5-LOX in fibroblast-like synoviocytes (FLS) or knocking the 5LOX gene in a mouse model with RA decreased inflammatory cytokine expression and paw inflammation (61). The BLT2 receptor also appears to be involved in the pathogenesis of RA, as it was shown to mediate LTB4-induced upregulation of TNF and IL1β in FLS (62), and BLT2 deficient mice presented reduced incidence and severity of arthritis in an animal model with RA (63). Intraarticular glucocorticoids decreased the expression of 5-LOX in the synovial tissue (57) and methotrexate decreased LTB4 secretion in polymorphonuclear cells from RA patients (64, 65). Interestingly, no relevant clinical effects were observed in RA patients treated with Zileuton, a 5-LOX inhibitor (66, 67). 15-LOX is also expressed in RA synovium (58), but the studies on this pathway have reported contradictory results in RA (68).

There is less information available in arthritis about the role of other AA-derived oxylipins such as the hydroxyeicosatetraenoic acids (HETE) or other lipids derived *via* the CYP450 pathways, such as epoxyeicosatrienoic acid (EETs), which have been proposed to have anti-inflammatory properties.

Finally, another group of PUFA-derived lipids are the specialized pro-resolving mediators (SPM), which are essential for the resolution of inflammation. They include lipoxins (derived from AA), maresins (derived from DHA), and resolvins (derived from EPA and DHA) (69). Several studies in RA patients and in animal models of arthritis suggest a role for these oxylipins in arthritis. Lipoxins (LX) are generated by the combined action of 5-LOX and 15-LOX-1 (an isoform of 15-LOX). LXA4 has been detected in RA SF (58) and is known to inhibit neutrophil chemotaxis, adhesion and migration, which could result in less articular damage (70). No human studies are available, but arthritis models induced in 12/15-LOX (an orthologue of human 15-LOX-1) deficient mice showed enhanced joint inflammation and destruction and were associated with low levels of LXA4 in the synovial extracts (71). Moreover, treatment of another animal model (CIA) with LXA4 agonists significantly decreased clinical and histological scores of arthritis (72).

In another study, resolvin (Rv) RvD3 was reduced in serum from RA patients compared to controls, and administration of RvD3 reduced joint leukocytes as well as paw joint oxylipins, clinical scores, and edema in the mouse model (73). RvD3 levels were also reduced in inflamed joints from mice with delayed-resolving arthritis when compared to joints with self-resolving inflammatory arthritis. These data suggest a possible therapeutic role of RvD3 in RA (73). RvD1 was also found to be decreased in serum of RA patients compared to controls, and its administration in the CIA mouse model decreased inflammation as well as cartilage damage. Furthermore, *in vitro* studies showed that RvD1 decreased migration and proliferation of RA FLS, properties that are associated with disease progression (74–76). RvD5 is another SPM that was shown to decrease inflammation in a mouse model of RA (77). Of interest, a recent study described macrophage synovial subpopulations (MerTK^pos^TREM2^high^ and MerTK^pos^LYVE1^pos^) with a unique remission transcriptomic signature enriched in negative regulators of inflammation in RA synovial tissue. MerTKpos synovial macrophages from RA patients in remission produce a higher amount of SPMs, including RvD1, which suggests they may promote resolution of inflammation (78). All these results suggest that these pathways are critical for arthritis pathogenesis, but we need a better understanding of their role to identify critical therapeutic targets.

### Sphingosine-1-Phosphate Pathway

Sphingosine-1-phosphate (S1P) is another lipid that has been studied in RA and acts on a series of tissue receptors (S1P1 to 3). Recently, a study found an increased concentration of S1P in SF of RA patients compared to controls (79). Ceramide was found to be a potent inducer of apoptosis of proliferative RA FLS *in vitro* and *in vivo*, suggesting that this lipid messenger might inhibit synovial proliferation (80). *In vitro* studies showed that the addition of C2-ceramide was able to inhibit platelet-derived growth factor (PDGF)-induced cell cycle progression of RA FLS, by the inhibition of anti-apoptotic kinases, such as Akt and ERK1/2, which suggests that the inhibition of these kinases may contribute to the apoptotic effects of ceramide by eliminating proliferative signals in the rheumatoid synovium (81). Studies performed in animal models also provide support for the involvement of the S1P pathway in synovitis. S1P receptor is upregulated in synovial tissue from the CIA mouse model, and inflammation increases S1P/S1P3 signaling, which stimulates increased production of interleukin (IL)-6 in FLS from CIA mice. Additionally, S1P3 receptor KO mice developed a lower degree of arthritis compared to *wild type* mice (82). Moreover, proangiogenic factors can stimulate the sphingosine kinase 1 (SphK1)/S1P/S1P1 pathway to upregulate proliferation and migration and facilitate angiogenesis in a rat model with RA (83). Hence, the data suggest that S1P could be a potential therapeutic target in RA, although further studies are needed to establish its role in RA pathogenesis.

### Other Lipids

Regarding other types of lipids involved in arthritis, older studies revealed that disease progression in the CIA mouse model was associated with a significant reduction in the expression of genes involved in lipogenesis (INSIG1, SREBP1a and ACC) and lipid accumulation (DGAT1, DGAT2, PLIN1 and PLIN2) (84). RA susceptibility genes (TRAF1/C5, STAT4 and HLA-DRB1-SE) might also be involved in the regulation of lipid metabolism (85). Other RA susceptibility genes, specifically FADS1 and 2 and BLK (BLK Proto-Oncogene, Src Family Tyrosine Kinase), are part of FA metabolism (86–90). Finally, several single nucleotide polymorphisms (SNP) in genes involved in lipid metabolism have been described to be associated with RA. SLC22A4, a transporter related to isovaleryl/carnitine (involved in lipid transportation), was associated with RA in a Japanese (91), but not in a Canadian population (92). In another study, Geiger et al. (93) also described 2 SNPs, rs9309413 and rs4775041, on PLEK (Pleckstrin) and LIPC (Hepatic Triacylglycerol Lipase) genes, which are related to sphingomyelin and PE synthesis respectively, that were associated with risk of RA in a previous study (94). Finally, DLG2 (Disks Large MAGUK Scaffold Protein 2), a gene associated with GPL metabolism (95), was described to be related to the response to TNF inhibitors in RA patients (96).

### Lipids in Serum

Several studies have also determined lipids in the serum of patients with rheumatic diseases. We have summarized these studies in Table 3. In one of these studies, Gomez et al. found an upregulation of various SPM in peripheral blood from patients with a pauci-immune-fibroid pathotype (characterized by histologic analysis of synovial tissue from biopsies). They also found different lipid profiles were associated with response to DMARDs, suggesting a still underdeveloped understanding of these mediators in arthritis (102).

**Table 3:** Lipidomic studies in serum or plasma in individuals with arthritis and controls.

**Disease**	**Patients**	**Type of sample**	**Types of lipids**	**Findings**
EORA and PMR	44 EORA 20 PMR 18 controls	Serum	Oxylipins	- The ratio of n-3/n-6 PUFA was significantly downregulated in EORA, but not in PMR patients, as compared to controls, and increased after treatment. - Two oxylipins, 4-HDoHE and 8,15-diHETE differentiated both diseases (97).
RA	60 early RA 11 arthralgia 28 controls	Serum	Oxylipins	- Different oxylipins profiles were identified across the stages of arthralgia and early RA. - Different oxylipin profile were observed in patients with more severe disease and who were less likely to achieve remission (98).
RA	32 active RA 33 RA in remission	Serum	SPMs	- SPM concentrations (LXA4, RvD1, and RvE1) were higher in sera of RA patients with active disease compared to remission (99).
RA	78 RA	Serum	Lipid composition carnitine- and choline- derivatives	- Higher total FA and total cholesterol concentrations were found in active RA. - Elevated PL concentrations with lower choline, elevated medium-chain acylcarnitines (MC-AC), and decreased ratios of MC-AC and long-chain (LC)-AC were associated with prednisolone medication (100).
RA	255 RA 100 controls	Serum	GPL, GL, Carnitines	- Acyl carnitines (20:3), PE (18:1), and LPE (20:3) correlated with RA disease activity. - PA (28:0) negatively correlated with RA disease activity (101).
RA	30 RA responders to DMARDs 24 non-responders	Plasma	Oxylipins	- Upregulation of SPMs and pro-inflammatory and immunosuppressive mediators including PGD2 and TXB2 in patients with a pauci-immune-fibroid pathotype (characterized by histologic analysis of synovial tissue from biopsies). - Different lipid profiles were associated to response to DMARDs (102).
Pre-RA	30 pre-RA 19 controls	Plasma	GPLs, GLs	- The majority of PL and SM were higher in pre-RA in comparison with controls (103).
PsA	41 PsA	Serum	Oxylipins	- Pro-inflammatory oxylipins such as PGE2, HXB3 or 6,15-dk, dh, PGF1a, and EPA-derived oxylipins, such as 11-HEPE, 12-HEPE and 15-HEPE correlated with joint disease score. - RvD1 was down-regulated in patients with high disease activity (104).
PsO and PsA	20 PsO 19 PsA	Serum	Oxylipins	- PsO and PsA patients with higher PASI score had lower serum AA-derived oxylipins. - AA-derived oxylipins (5,15 di-HETE 5-oxoETE, PGE2, 11bPGE2, and LTB4 were associated with enthesitis (105).
PsA	20 PsO who develop PsA 30 PsO with no PsA 10 controls	Serum	Untargeted	- Elevated levels of selected LCFA (e.g., 3-hydroxytetradecanedioic acid) in severe PsA. - 1,11-undecanedicarboxylic acid was identified as a classifier in PsA patients - Oxylipins were detected solely in moderate and severe PsA (106).
OA	49 early OA 43 late OA	SF and serum	GLs, GPLs, SPs	- The lipid levels were 4–10-fold higher in serum than in SF - With advanced disease stage more lipid species are found at elevated serum levels as compared to normal controls (107).
OA	23 late OA 6 controls	SF	FA	- The n-6/n-3 ratio was significantly lower in the OA group. - AA concentrations were lower in OA SF, while tetracosadienoic acid and nervonic acid (MUFAs) were higher in OA SF (108).

*RA, rheumatoid arthritis; EORA, elderly onset rheumatoid arthritis; PMR, polymyalgia rheumatica; PsA, psoriatic arthritis; PsO, psoriasis; OA, osteoarthritis; SF, synovial fluid; AA, arachidonic acid; PL, phospholipids; LCFA, long chain fatty acids; FA, fatty acids; PUFA, polyunsaturated fatty acids; MUFA, monounsaturated fatty acids; MTX, methotrexate; SPM, specialized pro-resolving mediators; LC-AC, long chain acylcarnitines; SM, sphingomyelins; DMARDs, disease modifying anti-rheumatic drugs; PA, phosphatidic acid; PE, Phosphoethanolamine; LPE, lysophosphoethanolamine; SPs, sphingolipids; GLs: glycerolipids; GPLs, glycerophospholipids*.

Overall, human observational studies and mechanistic *in vitro* and animal studies offer evidence for the alteration of lipid metabolism in arthritis, with the presence of an imbalance between pro- and inflammatory lipids. The lack of lipidomic data in synovial tissue prompted us to review applications of tissue lipidomics in other diseases and identify inflammation-related changes, which might also play a role in arthritis.

## Lipidomics in Other Inflammatory Diseases

Most of the studies that have performed tissue lipidomics come from the fields of dermatology and inflammatory bowel disease, since the diagnostic process involves a biopsy. Most of the studies offer lipidomic profiles in the different tissues compared to controls, some of them attempt correlations with disease activity, however, in a large number of cases, the functional role of the lipids described in those studies is not known.

### Inflammatory Bowel Disease (IBD)

Lipidomics has been used in the field of IBD to better understand disease pathogenesis, as well as to identify biomarkers of diagnosis, disease activity, and response to treatment. The available studies describe the lipidomic profile in the intestinal mucosa of both ulcerative colitis (UC) and Crohn's disease (CD) compared to mucosa of controls, although a big limitation of all these studies is their small sample size.

#### Phospholipids

Analyzing ileal biopsy samples from CD patients with quiescent disease, Sewell et al. (109) reported a significant reduction in phosphatidylinositol (PI) 16:0/18:1 (as a percentage of total PI) in CD compared to controls, whose synthesis was also decreased in peripheral blood monocyte derived macrophages isolated from CD patients. PI is part of the PL classes, which are important components of the intestinal mucus as well as the membranes of cells which contribute to the protective effect of the intestinal barrier (110). Hence, the alteration in PI could contribute to both damage of the mucosal barriers function as well as an imbalance in the secretion of pro-inflammatory cytokines in CD patients. Another group (111) also compared lipid content in the colonic mucosa of patients with UC and reported that several PCs and PEs, mainly PE(38:3), were elevated in UC patients with active disease compared to remission, as well as in remission compared to contorosl. PE has a role in apoptosis in TNF induced inflammation (112, 113).

#### Polyunsaturated Fatty Acids

Pearl and colleagues (114) measured the esterified and non-esterified bioactive PUFA in gut mucosal biopsies from patients with quiescent, active UC, and from matched controls. They had also paired samples of inflamed- non-inflamed mucosa. AA, docosapentaenoic acid (DPA) and DHA were significantly higher, and LA, α-LNA and EPA were significantly lower in inflamed compared to non-inflamed mucosa, but also in inflamed mucosa of patients with UC compared to mucosa of controls. The comparison of PUFA in non-inflamed mucosa from UC patients with age–sex matched controls did not show significant differences except for DPA, which was significantly lower in non-inflamed mucosa. Importantly, the mass % of AA and DPA positively correlated with both endoscopically and histological disease activity, while the mass % of a-LNA and EPA negatively correlated with the same parameters of disease activity. These changes were also observed in treatment naïve patients. The findings in this study suggest an imbalance of n-3 and n-6 PUFA, with an increase of AA availability, which is a precursor of pro-inflammatory oxylipins, and a decrease in EPA, which is a precursor of anti-inflammatory oxylipins.

#### Oxylipins

Concentrations of 5-HETE, 12-HETE and 15-HETE (AA derived oxylipins *via* the LOX pathway), PGE2, PGD2, and TXB2 (AA derived oxylipins *via* COX pathway), as well as 11-HETE (AA derived oxylipin *via* non-enzymatic pathway) in UC inflamed mucosa were significantly higher than in adjacent non-inflamed mucosa. Moreover, these mediators also correlated with the level of inflammation measured by histology. Of those metabolites, only PGE2, PGD2, TXB2, and 15-HETE were confirmed in inflamed mucosa from treatment naïve patients (115).

Oxylipins and endocannabinoids (eCBs) were also studied by Diab et al. (116) in UC, comparing UC treatment naïve patients, with patients in remission, and with controls. They reported that patients with active disease presented a significant elevation in concentrations of n-6 AA–derived oxylipins, specifically, PGE2, TXB2, trans-LTB4, and 12-HETE, in addition to lower concentrations of n-3 eCBs (docosahexaenoyl ethanolamide and eicosapentaenoyl ethanolamide). Only 15(s)-HETrE, an AA derived oxylipin, was higher in mucosa of patients in remission compared to controls. 15-HETrE has anti-inflammatory properties and could be involved in maintaining the remission state. The results of this study also support the idea of an imbalance between pro- and anti-inflammatory oxylipins in the inflammatory process underlying UC disease. An interesting finding was the decrease in eCBs, which also negatively correlated with pro-inflammatory cytokines suggesting an anti-inflammatory role.

#### Sphingolipids

Bazarganipour and colleagues (117) applied targeted lipidomics to colonic inflamed tissues compared to non-inflammatory tissue from the same patients with different severity of CD (in remission, mild or moderate/severe disease), and who also received different treatments. The levels of sphinganine (dhSph) and most dihydroceramides (dhCer) were significantly decreased in inflamed tissue, suggesting that the *de novo* synthesis of SPs is reduced in inflamed tissue. In IBD, the *de novo* synthesis of SPs is considered critical for the integrity of the epithelial barrier, whose disruption is associated with intestinal inflammation and bacterial invasion (118). The decrease was not due to a decrease in the expression of the enzymes serine palmitoyltransferase (SPT) and ceramide synthases (CerS), involved in dhCer synthesis, so the authors suggest it could be due to post-translational modifications of these enzymes. They did however notice an enhanced expression in CerS3 in the lamina propria, by both mRNA and IHC, suggesting it could represent the result of invaded immune cells. Additionally, the concentrations of C16:0- and C24:0-lactosyl-ceramide (LacCer) increased in inflamed tissue in comparison to control tissue. LacCer functions as pattern-recognition receptor in human cells and activates an innate immune response (119, 120). Therefore, the increase in LacCer in inflamed colon tissue would increase binding of pathogens, enhancing the immune response and inflammation.

Ceramides were associated with bowel inflammation in another study (111), which reported that Cer(d18:1/24:2) and Cer(d18:1/24:0) increased from remission to active inflammation in UC patients, and SF of RA and OA patients contained higher levels of Cer(d18:1/24:2) and Cer(d18:1/24:0).

As presented above, some of them, such as the imbalance between n-3 and n-6 derived oxylipins and sphingolipids, may be relevant in synovitis so would be worth analyzing in inflamed synovium.

### Skin Diseases

Lipids are essential components of the skin and play a critical role in maintaining the skin barrier. Lipidomics has been used in several studies of skin diseases such as psoriasis and atopic dermatitis (AD).

#### Sphingolipids

The level of *de novo* ceramides synthesis, the protein expression of SPT (serine palmitoyltransferase, the enzyme involved in ceramide synthesis), and the number of ceramides were described to be significantly lower in psoriatic plaques compared to the non-lesional epidermis [reviewed here (121)]. This data is also supported by animal studies, since SPT knock out mice develop skin psoriasis and have low skin levels of ceramides. Interestingly, the percentage reductions of both—ceramide synthesis and its epidermal level—were positively correlated with the Psoriasis Area and Severity Index (PASI) score in mild to moderate psoriasis (122, 123).

Another study (124) used lipidomics to measure both circulating and skin lipids in psoriasis patients, lesional, and non-lesional skin. The lipid species that were analyzed include non-hydroxylated fatty acid/sphingosine (NS) class of sphingolipids, with an extensive coverage of the SP pathway (30 species were quantified in total), consisting of a range of compounds including sphingomyelins, ceramides, hexosylceramides, lactosylceramides, and dihydroceramides with varying FA chain lengths. The analysis also included free phosphorylated and non-phosphorylated NS sphingoid bases [sphingosine, sphinganine, S1P, and sphinganine-1-phosphate (Spa1P)]. Increased levels (*P* < 0.001) for most of the ceramides were observed in lesional skin relative to non-lesional and control skin. Levels of sphingomyelins were altered in lesional skin in a FA chain length-dependent manner with increases in C16:0-, C24:1- and C24:0-sphingomyelins. This observation is interesting since in cancer a higher content in lipids with longer chains and increased number of unsaturated bonds is associated with a more flexible phenotype of the cells, allowing for increased proliferation and invasion.

#### Glycerophospholipids

Another study (125) performed in atopic dermatitis (AD) compared metabolomic profiles of lesional skin (AD-L) and non-lesional skin (AD-NL) with the skin of controls. The quantified metabolites, including SM and PC, are sources of bioactive compounds that are involved in different signaling pathways. They found 40 PCs that had elevated ratios in AD-L skin compared to AD-NL skin, and 6 PCs that had higher concentrations in AD-L skin compared to both AD-NL and C skin. As in psoriasis, the concentrations of 4 lysoPCs, which are derived from PCs, were increased in AD lesional skin compared to non-lesional skin and they hypothesized that one of their roles could be the attraction of T lymphocytes to the skin.

#### Oxylipins

In psoriatic lesions, the levels of unsaturated FAs differ significantly. All products of LOX are abundant and involve monohydroxy derivatives from AA [5-, 8-, 9-, 11-, 12-, and 15-hydroxyeicosatetraenoic acid (HETE)] and from LA [9- and 13-hydroxyoctadecadienoic acid (HODE)] (126). These lipids have specific physiological functions in the epidermis. For example, 13-HODE is thought to have anti-inflammatory effects and the ability to maintain normal cell proliferation, as was shown in human and animal keratinocytes (127, 128). 9-HODE promotes the release of inflammatory cytokines (126, 129). However, the amount of 13-HODE produced by the psoriatic epidermis is not sufficient to inhibit the hyperproliferation of keratinocytes. Similarly, 12-HETE is a proinflammatory chemotactic agent (130), whereas 15-HETE reduces inflammatory cell infiltration. However, 15-HETE is higher in psoriatic lesions than 12-HETE (126). LOX oxidation products are further oxidized to produce epoxides, such as epoxy octadecadienoic acid and epoxyeicosatrienoic acid. These epoxides may promote neutrophil infiltration and inflammation (131, 132).

A recent study (133) found marked changes in both PL and oxylipin synthesis in psoriatic skin. This includes abundant AA metabolites, DHA and oxidized-DHA products, and PCs, and decreased PE, LPC, and resolvin D1, and are consistent with previous findings (126, 130). Lipid mediators can serve as both activators and suppressors of inflammation to elicit local effects (104). For example, LTB4 and 12-HETE (134) act as chemoattractant for neutrophils and macrophages in the skin. In contrast, 15-HETE acts as a negative regulator in LTB4- and 12-HETE–induced inflammation (135). DHA affects skin homeostasis by activating keratinocytes to express proinflammatory mediators. In addition, resolvin D1 is decreased in psoriatic skin and downregulated by phospholipase A2, exerts a protective role in psoriasis-like dermatitis and other types of inflammatory responses (136, 137).

**Figure 5:**
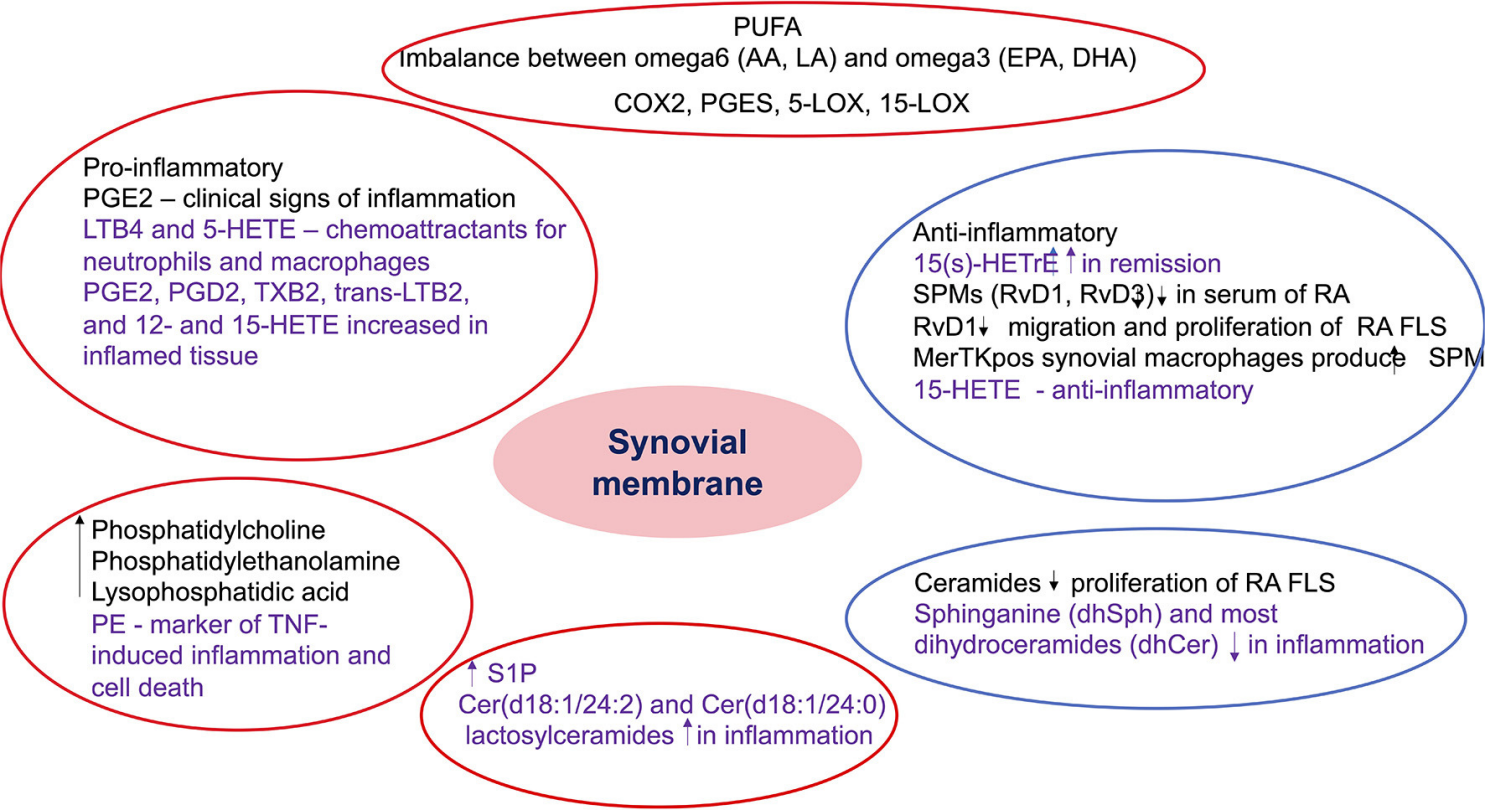
Summary of the role of bioactive lipids in synovial pathology. The findings included in the red circles include lipids with potential pro-inflammatory role, while the ones included in the blue circles include anti-inflammatory and pro-resolving metabolites. The black font describes results from studies in inflammatory arthritis, while the purple font describes results from studies in non-rheumatic diseases but with potential role in inflammatory arthritis. PUFA, polyunsaturated fatty acids; AA, arachidonic acid; LA, linoleic acid; EPA, eicosapentaenoic acid; DHA, docosahexaenoic acid; COX, cyclooxygenase; PGES, prostaglandin E synthase; LOX, lipoxygenase; PG, prostaglandin E; HETE, hydroxyeicosatetraenoic acid; LT, leukotriene; TX, thromboxane; HETrE, hydroxyeicosatrienoic acid; SPM, specialized proresolving mediators; Rv, resolvin; RA, rheumatoid arthritis; FLS, fibroblast-like synoviocytes; PE, phosphatidylethanolamine; TNF, tumor necrosis factor; S1P, sphingosine 1 phosphate; Cer, ceramides.

The studies that we reviewed in other tissues show evidence of the role of different types of lipids (fatty acids, oxylipins, phospholipids) not only in the pathogenesis of these diseases but also in predicting response to treatment. The application of lipidomics to the study of synovial tissue may help to assess whether these lipids are also altered or contribute to the inflammatory process in inflammatory arthritis (Table 4; Figure 5), paving the way for the discovery of new therapeutic targets and biomarkers of response to treatment.

**Table 4:** Biological role of lipids in the synovium of inflammatory arthritis.

**Lipid**	**Role**
GPL	- Elevated levels of PC, FAs and lysophosphatidic acids, and lower levels of lysophosphatidylcholines (LysoPC) in OA synovium compared to control tissue - The spatial distribution of specific GPLs correlates with hypertrophic, inflamed and vascularized synovial areas (28).
PE and PC	- PE and PC were higher in PsA synovium and SF compared to RA (37). - PE spatial distribution was associated with areas of the sublining layer with increased vascularity and inflammatory cell infiltrates (37).
Oxylipins	- COX2 and PGEs, involved in oxylipins synthesis, are over-expressed in RA, ankylosing spondylitis, and PsA synovium compared to OA (40–43). - PGE2 has a role in inflammation and pain (49, 50). - Inhibition of 5LOX in FLS decreased inflammatory cytokine expression (61). - RvD3 (73) and RvD1 was reduced in serum from RA patients compared to controls and *in vitro* studies showed RvD1 decreased migration and proliferation of RA FLS, properties that are associated with disease progression (74–76). - MerTKpos synovial macrophages from RA patients in remission produce a higher amount of SPMs, including RvD1, which suggest they may promote resolution of inflammation (78).
S1P	- S1P is increased in SF of RA patients compared to controls (79). - Ceramide is a potent inducer of apoptosis of proliferative RA FLS *in vitro* and *in vivo* (80). - C2-ceramide inhibits platelet-derived growth factor (PDGF)-induced cell cycle progression of RA FLS, hence decreasing proliferation (81).

*GPL, Glycerophospholipids; PE, Phosphatidylethanolamine; PE, Phosphatidylcholine; FA, fatty acid; PUFA, Polyunsaturated fatty acids; S1P, Sphingosine-1-phosphate; RA-FLS, Rheumatoid Arthritis-Fibroblast like synoviocytes; RA, rheumatoid arthritis; PsA, psoriatic arthritis; OA, osteoarthritis; SF, synovial fluid; COX2, cyclooxygenase 2; PGE2, prostaglandin E2; LOX, lipoxygenase; Rv, Resolvin; SPM, specialized pro-resolving mediators*.

## Conclusion

The characterization of the cells in the synovial membrane is now being actively pursued in RA as part of the Accelerating Medicines Partnership consortium (17, 138). Combining this data with lipidomic cell signatures could provide useful information to not only better understand the role of each type of cell, and functional mediators, but also identify biomarkers of disease activity or response to treatment. In addition, the characterization of the ratio n6-n3 PUFA and the quantification of pro-, anti-inflammatory and pro-resolving mediators, among other lipid subtypes, in the synovial tissue, would offer more information on the involvement of these bioactive lipids in the arthritis pathogenesis.

## Author Contributions

RC, JM-S, AS, AK, and MG have contributed to the literature review. All authors were involved in drafting the article or revising it critically for important intellectual content, and approved the final version to be published.

## Funding

Supported by grants from the National Institutes of Health: R01 AR073324 to MG, T32AR064194 to RC and JM-S, and Pfizer ASPIRE research grant and Novartis IIT to AK, AS, and MG.

## Conflict of Interest

The authors declare funding from Pfizer and Novartis. Pfizer and Novartis had no role in study design, data collection and analysis, decision to publish, or preparation of the manuscript.

## Publisher's Note

All claims expressed in this article are solely those of the authors and do not necessarily represent those of their affiliated organizations, or those of the publisher, the editors and the reviewers. Any product that may be evaluated in this article, or claim that may be made by its manufacturer, is not guaranteed or endorsed by the publisher.

## References

[1] Smith MD. The normal synovium. Open Rheumatol J. (2011) 5:100–6. 10.2174/1874312901105010100 22279508 PMC3263506

[2] Hitchon CA El-Gabalawy HS. The synovium in rheumatoid arthritis. Open Rheumatol J. (2011) 5:107–14. 10.2174/1874312901105010107 22279509 PMC3263474

[3] Bartok B Firestein GS. Fibroblast-like synoviocytes: key effector cells in rheumatoid arthritis. Immunol Rev. (2010) 233:233–55. 10.1111/j.0105-2896.2009.00859.x 20193003 PMC2913689

[4] Celis R Cuervo A Ramirez J Canete JD. Psoriatic synovitis: singularity and potential clinical implications. Front Med. (2019) 6:14. 10.3389/fmed.2019.00014 30805340 PMC6378889

[5] Reece RJ Canete JD Parsons WJ Emery P Veale DJ. Distinct vascular patterns of early synovitis in psoriatic, reactive, and rheumatoid arthritis. Arthritis Rheum. (1999) 42:1481–4.3. 10403277 10.1002/1529-0131(199907)42:7<1481::AID-ANR23>3.0.CO;2-E

[6] van de Sande MG Baeten DL. Immunopathology of synovitis: from histology to molecular pathways. Rheumatology. (2016) 55:599–606. 10.1093/rheumatology/kev330 26359330

[7] Chen S Noordenbos T Blijdorp I van Mens L Ambarus CA Vogels E. Histologic evidence that mast cells contribute to local tissue inflammation in peripheral spondyloarthritis by regulating interleukin-17A content. Rheumatology. (2019) 58:617–27. 10.1093/rheumatology/key331 30517745

[8] Sanchez-Lopez E Coras R Torres A Lane NE Guma M. Synovial inflammation in osteoarthritis progression. Nat Rev Rheumatol. (2022) 1:18. 10.1038/s41584-022-00749-9 35165404 PMC9050956

[9] Katchamart W Narongroeknawin P Chanapai W Thaweeratthakul P. Health-related quality of life in patients with rheumatoid arthritis. BMC Rheumatol. (2019) 3:34. 10.1186/s41927-019-0080-9 31428740 PMC6694487

[10] Gladman DD. Clinical features and diagnostic considerations in psoriatic *Arthritis*. Rheum Dis Clin North Am. (2015) 41:569–79. 10.1016/j.rdc.2015.07.003 26476219

[11] Genitsaridi I Flouri I Plexousakis D Marias K Boki K Skopouli F. Rheumatoid arthritis patients on persistent moderate disease activity on biologics have adverse 5-year outcome compared to persistent low-remission status and represent a heterogeneous group. Arthritis Res Ther. (2020) 22:226. 10.1186/s13075-020-02313-w 32993800 PMC7523072

[12] Ajeganova S Huizinga T. Sustained remission in rheumatoid arthritis: latest evidence and clinical considerations. Ther Adv Musculoskelet Dis. (2017) 9:249–62. 10.1177/1759720X17720366 28974987 PMC5613855

[13] Mandelin AM 2nd Homan PJ Shaffer AM Cuda CM Dominguez ST. Transcriptional profiling of synovial macrophages using minimally invasive ultrasound-guided synovial biopsies in rheumatoid arthritis. Arthritis Rheumatol. (2018) 70:841–54. 10.1002/art.40453 29439295 PMC5984677

[14] Saraiva F. Ultrasound-guided synovial biopsy: a review. Front Med. (2021) 8:632224. 10.3389/fmed.2021.632224 33968950 PMC8100029

[15] Humby F Lewis M Ramamoorthi N Hackney JA Barnes MR Bombardieri M. Synovial cellular and molecular signatures stratify clinical response to csDMARD therapy and predict radiographic progression in early rheumatoid arthritis patients. Ann Rheum Dis. (2019) 78:761–72. 10.1136/annrheumdis-2018-214539 30878974 PMC6579551

[16] Mizoguchi F Slowikowski K Wei K Marshall JL Rao DA Chang SK. Functionally distinct disease-associated fibroblast subsets in rheumatoid arthritis. Nat Commun. (2018) 9:789. 10.1038/s41467-018-02892-y 29476097 PMC5824882

[17] Kuo D Ding J Cohn IS Zhang F Wei K Rao DA. HBEGF. Sci Transl Med. (2019) 11:eaau8587. 10.1126/scitranslmed.aau8587 31068444 PMC6726376

[18] Nerviani A Di Cicco M Mahto A Lliso-Ribera G Rivellese F Thorborn G. A pauci-immune synovial pathotype predicts inadequate response to TNFα-blockade in rheumatoid arthritis patients. Front Immunol. (2020) 11:845. 10.3389/fimmu.2020.00845 32431716 PMC7214807

[19] Zhang C Wang K Yang L Liu R Chu Y Qin X. Lipid metabolism in inflammation-related diseases. Analyst. (2018) 143:4526–36. 10.1039/C8AN01046C 30128447

[20] Leuti A Fazio D Fava M Piccoli A Oddi S Maccarrone M. Bioactive lipids, inflammation and chronic diseases. Adv Drug Deliv Rev. (2020) 159:133–69. 10.1016/j.addr.2020.06.028 32628989

[21] Robinson G Pineda-Torra I Ciurtin C Jury EC. Lipid metabolism in autoimmune rheumatic disease: implications for modern and conventional therapies. J Clin Invest. (2022) 132:e148552. 10.1172/JCI148552 35040437 PMC8759788

[22] Yang K Han X. Lipidomics: techniques, applications, and outcomes related to biomedical sciences. Trends Biochem Sci. (2016) 41:954–69. 10.1016/j.tibs.2016.08.010 27663237 PMC5085849

[23] Apffel A Zhao L Sartain MJ. A novel solid phase extraction sample preparation method for lipidomic analysis of human plasma using liquid chromatography/mass spectrometry. Metabolites. (2021) 11:294. 10.3390/metabo11050294 34064397 PMC8147762

[24] Wolrab D Chocholoušková M Jirásko R Peterka O Holčapek M. Validation of lipidomic analysis of human plasma and serum by supercritical fluid chromatography-mass spectrometry and hydrophilic interaction liquid chromatography-mass spectrometry. Anal Bioanal Chem. (2020) 412:2375–88. 10.1007/s00216-020-02473-3 32078000

[25] Quehenberger O Armando AM Brown AH Milne SB Myers DS Merrill AH. Lipidomics reveals a remarkable diversity of lipids in human plasma. J Lipid Res. (2010) 51:3299–305. 10.1194/jlr.M009449 20671299 PMC2952570

[26] Hartler J Armando AM Trötzmüller M Dennis EA Köfeler HC Quehenberger O. Automated annotation of sphingolipids including accurate identification of hydroxylation sites using MS. Anal Chem. (2020) 92:14054–62. 10.1021/acs.analchem.0c03016 33003696 PMC7581017

[27] Quehenberger O Armando A Dumlao D Stephens DL Dennis EA. Lipidomics analysis of essential fatty acids in macrophages. Prostaglandins Leukot Essent Fatty Acids. (2008) 79:123–9. 10.1016/j.plefa.2008.09.021 18996688 PMC2643973

[28] Rocha B Cillero-Pastor B Ruiz-Romero C Paine MRL Cañete JD Heeren RMA. Identification of a distinct lipidomic profile in the osteoarthritic synovial membrane by mass spectrometry imaging. Osteoarthritis Cartilage. (2021) 29:750–61. 10.1016/j.joca.2020.12.025 33582239

[29] Fahy E Cotter D Sud M Subramaniam S. Lipid classification, structures and tools. Biochim Biophys Acta. (2011) 1811:637–47. 10.1016/j.bbalip.2011.06.009 21704189 PMC3995129

[30] Kimura T Jennings W Epand RM. Roles of specific lipid species in the cell and their molecular mechanism. Prog Lipid Res. (2016) 62:75–92. 10.1016/j.plipres.2016.02.001 26875545

[31] Jakobsson A Westerberg R Jacobsson A. Fatty acid elongases in mammals: their regulation and roles in metabolism. Prog Lipid Res. (2006) 45:237–49. 10.1016/j.plipres.2006.01.004 16564093

[32] Steensels S Ersoy BA. Fatty acid activation in thermogenic adipose tissue. Biochim Biophys Acta Mol Cell Biol Lipids. (2019) 1864:79–90. 10.1016/j.bbalip.2018.05.008 29793055

[33] Li M Fan P Wang Y. Lipidomics in health and diseases-beyond the analysis of lipids. J Glycom Lipidomics. (2015) 5:1. 10.4172/2153-0637.1000126

[34] Harrieder EM Kretschmer F Bocker S Witting M. Current state-of-the-art of separation methods used in LC-MS based metabolomics and lipidomics. J Chromatogr B Analyt Technol Biomed Life Sci. (2022) 1188:123069. 10.1016/j.jchromb.2021.123069 34879285

[35] Coras R Murillo-Saich JD Guma M. Circulating pro- and anti-inflammatory metabolites and its potential role in rheumatoid arthritis pathogenesis. Cells. (2020) 9:827. 10.3390/cells9040827 32235564 PMC7226773

[36] Dayer JM Krane SM Russell RG Robinson DR. Production of collagenase and prostaglandins by isolated adherent rheumatoid synovial cells. Proc Natl Acad Sci USA. (1976) 73:945–9. 10.1073/pnas.73.3.945 176663 PMC336037

[37] Rocha B Cillero-Pastor B Illiano A Calamia V Pinto G Amoresano A. Distinct lipidomic signatures in synovium and synovial fluid of patients with rheumatoid arthritis versus psoriatic arthritis.*Arthritis Rheumatol*.(2021) 73 (suppl 10). Available online at: https://acrabstracts.org/abstract/distinct-lipidomic-signatures-in-synovium-and-synovial-fluid-of-patients-with-rheumatoid-arthritis-versus-psoriatic-arthritis/

[38] Masuda S Murakami M Komiyama K Ishihara M Ishikawa Y Ishii T. Various secretory phospholipase A2 enzymes are expressed in rheumatoid arthritis and augment prostaglandin production in cultured synovial cells. FEBS J. (2005) 272:655–72. 10.1111/j.1742-4658.2004.04489.x 15670148

[39] Hulkower KI Wertheimer SJ Levin W Coffey JW Anderson CM Chen T. Interleukin-1 beta induces cytosolic phospholipase A2 and prostaglandin H synthase in rheumatoid synovial fibroblasts. Evidence for their roles in the production of prostaglandin E2. Arthritis Rheum. (1994) 37:653–61. 10.1002/art.1780370508 8185692

[40] Crofford LJ Wilder RL Ristimäki AP Sano H Remmers EF Epps HR. Cyclooxygenase-1 and−2 expression in rheumatoid synovial tissues. Effects of interleukin-1 beta, phorbol ester, and corticosteroids. J Clin Invest. (1994) 93:1095–101. 10.1172/JCI117060 8132748 PMC294048

[41] Kang RY Freire-Moar J Sigal E Chu CQ. Expression of cyclooxygenase-2 in human and an animal model of rheumatoid arthritis. Br J Rheumatol. (1996) 35:711–8. 10.1093/rheumatology/35.8.711 8761181

[42] Siegle I Klein T Backman JT Saal JG Nüsing RM Fritz P. Expression of cyclooxygenase 1 and cyclooxygenase 2 in human synovial tissue: differential elevation of cyclooxygenase 2 in inflammatory joint diseases. Arthritis Rheum. (1998) 41:122–9. 9433877 10.1002/1529-0131(199801)41:1<122::AID-ART15>3.0.CO;2-8

[43] Kojima F Kapoor M Kawai S Crofford LJ. New insights into eicosanoid biosynthetic pathways: implications for arthritis. Expert Rev Clin Immunol. (2006) 2:277–91. 10.1586/1744666X.2.2.277 20477078

[44] Korotkova M Westman M Gheorghe KR af Klint E Trollmo C Ulfgren AK. Effects of antirheumatic treatments on the prostaglandin E2 biosynthetic pathway. Arthritis Rheum. (2005) 52:3439–47. 10.1002/art.21390 16255020

[45] Stichtenoth DO Thoren S Bian H Peters-Golden M Jakobsson PJ Crofford LJ. Microsomal prostaglandin E synthase is regulated by proinflammatory cytokines and glucocorticoids in primary rheumatoid synovial cells. J Immunol. (2001) 167:469–74. 10.4049/jimmunol.167.1.469 11418684

[46] Brodie MJ Hensby CN Parke A Gordon D. Is prostacyclin in the major pro-inflammatory prostanoid in joint fluid? Life Sci. (1980) 27:603–8. 10.1016/0024-3205(80)90310-0 6999274

[47] Dorris SL Peebles RS Jr. PGI2 as a regulator of inflammatory diseases. Mediators Inflamm. (2012) 2012:926968. 10.1155/2012/926968 22851816 PMC3407649

[48] Basu S Whiteman M Mattey DL Halliwell B. Raised levels of F(2)-isoprostanes and prostaglandin F(2alpha) in different rheumatic diseases. Ann Rheum Dis. (2001) 60:627–31. 10.1136/ard.60.6.627 11350853 PMC1753663

[49] Hoxha M. A systematic review on the role of eicosanoid pathways in rheumatoid arthritis. Adv Med Sci. (2018) 63:22–9. 10.1016/j.advms.2017.06.004 28818745

[50] Trebino CE Stock JL Gibbons CP Naiman BM Wachtmann TS Umland JP. Impaired inflammatory and pain responses in mice lacking an inducible prostaglandin E synthase. Proc Natl Acad Sci USA. (2003) 100:9044–9. 10.1073/pnas.1332766100 12835414 PMC166435

[51] Maicas N Ibanez L Alcaraz MJ Ubeda A Ferrandiz ML. Prostaglandin D2 regulates joint inflammation and destruction in murine collagen-induced arthritis. Arthritis Rheum. (2012) 64:130–40. 10.1002/art.30656 21898357

[52] Sahap Atik O. Leukotriene B4 and prostaglandin E2-like activity in synovial fluid in osteoarthritis. Prostaglandins Leukot Essent Fatty Acids. (1990) 39:253–4. 10.1016/0952-3278(90)90002-3 2162064

[53] Wittenberg RH Willburger RE Kleemeyer KS Peskar BA. In vitro release of prostaglandins and leukotrienes from synovial tissue, cartilage, and bone in degenerative joint diseases. Arthritis Rheum. (1993) 36:1444–50. 10.1002/art.1780361017 8216404

[54] Kojima F Kato S Kawai S. Prostaglandin E synthase in the pathophysiology of arthritis. Fundam Clin Pharmacol. (2005) 19:255–61. 10.1111/j.1472-8206.2005.00316.x 15910650

[55] Gheorghe KR Sadique S Leclerc P Idborg H Wobst I Catrina AI. Limited effect of anti-rheumatic treatment on 15-prostaglandin dehydrogenase in rheumatoid arthritis synovial tissue. Arthritis Res Ther. (2012) 14:R121. 10.1186/ar3851 22616846 PMC3446502

[56] Liagre B Vergne P Rigaud M Beneytout JL. Expression of arachidonate platelet-type 12-lipoxygenase in human rheumatoid arthritis type B synoviocytes. FEBS Lett. (1997) 414:159–64. 10.1016/S0014-5793(97)00904-6 9305751

[57] Gheorghe KR Korotkova M Catrina AI Backman L af Klint E Claesson HE. Expression of 5-lipoxygenase and 15-lipoxygenase in rheumatoid arthritis synovium and effects of intraarticular glucocorticoids. Arthritis Res Ther. (2009) 11:R83. 10.1186/ar2717 19497113 PMC2714134

[58] Hashimoto A Hayashi I Murakami Y Sato Y Kitasato H Matsushita R. Antiinflammatory mediator lipoxin A4 and its receptor in synovitis of patients with rheumatoid arthritis. J Rheumatol. (2007) 34:2144–53. 17918787

[59] Chen M Lam BK Kanaoka Y Nigrovic PA Audoly LP Austen KF. Neutrophil-derived leukotriene B4 is required for inflammatory arthritis. J Exp Med. (2006) 203:837–42. 10.1084/jem.20052371 16567388 PMC2118292

[60] Kim ND Chou RC Seung E Tager AM Luster AD. A unique requirement for the leukotriene B4 receptor BLT1 for neutrophil recruitment in inflammatory arthritis. J Exp Med. (2006) 203:829–35. 10.1084/jem.20052349 16567386 PMC2118298

[61] Lin HC Lin TH Wu MY Chiu YC Tang CH Hour MJ. 5-Lipoxygenase inhibitors attenuate TNF-alpha-induced inflammation in human synovial fibroblasts. PLoS ONE. (2014) 9:e107890. 10.1371/journal.pone.0107890 25229347 PMC4168259

[62] Xu S Lu H Lin J Chen Z Jiang D. Regulation of TNFalpha and IL1beta in rheumatoid arthritis synovial fibroblasts by leukotriene B4. Rheumatol Int. (2010) 30:1183–9. 10.1007/s00296-009-1125-y 19809821

[63] Mathis SP Jala VR Lee DM Haribabu B. Nonredundant roles for leukotriene B4 receptors BLT1 and BLT2 in inflammatory arthritis. J Immunol. (2010) 185:3049–56. 10.4049/jimmunol.1001031 20656922

[64] Leroux JL Damon M Chavis C Crastes de Paulet A Blotman F. Effects of a single dose of methotrexate on 5- and 12-lipoxygenase products in patients with rheumatoid arthritis. J Rheumatol. (1992) 19:863–6. 1328631

[65] Sperling RI Benincaso AI Anderson RJ Coblyn JS Austen KF Weinblatt ME. Acute and chronic suppression of leukotriene B4 synthesis *ex vivo* in neutrophils from patients with rheumatoid arthritis beginning treatment with methotrexate. Arthritis Rheum. (1992) 35:376–84. 10.1002/art.1780350403 1314609

[66] Weinblatt ME Kremer JM Coblyn JS Helfgott S Maier AL Petrillo G. Zileuton, a 5-lipoxygenase inhibitor in rheumatoid arthritis. J Rheumatol. (1992) 19:1537–41. 1334515

[67] Diaz-Gonzalez F Alten RH Bensen WG Brown JP Sibley JT Dougados M. Clinical trial of a leucotriene B4 receptor antagonist, BIIL 284, in patients with rheumatoid arthritis. Ann Rheum Dis. (2007) 66:628–32. 10.1136/ard.2006.062554 17170051 PMC1954613

[68] Korotkova M Jakobsson PJ. Persisting eicosanoid pathways in rheumatic diseases. Nat Rev Rheumatol. (2014) 10:229–41. 10.1038/nrrheum.2014.1 24514915

[69] Serhan CN Levy BD. Resolvins in inflammation: emergence of the pro-resolving superfamily of mediators. J Clin Invest. (2018) 128:2657–69. 10.1172/JCI97943 29757195 PMC6025982

[70] Stables MJ Gilroy DW. Old and new generation lipid mediators in acute inflammation and resolution. Prog Lipid Res. (2011) 50:35–51. 10.1016/j.plipres.2010.07.005 20655950

[71] Krönke G Katzenbeisser J Uderhardt S Zaiss MM Scholtysek C Schabbauer G. 12/15-lipoxygenase counteracts inflammation and tissue damage in arthritis. J Immunol. (2009) 183:3383–9. 10.4049/jimmunol.0900327 19675173

[72] Zhang L Zhang X Wu P Li H Jin S Zhou X. BML-111, a lipoxin receptor agonist, modulates the immune response and reduces the severity of collagen-induced arthritis. Inflamm Res. (2008) 57:157–62. 10.1007/s00011-007-7141-z 18648754

[73] Arnardottir HH Dalli J Norling LV Colas RA Perretti M Serhan CN. Resolvin D3 is dysregulated in arthritis and reduces arthritic inflammation. J Immunol. (2016) 197:2362–8. 10.4049/jimmunol.1502268 27534559 PMC5011006

[74] Sun W Ma J Zhao H Xiao C Zhong H Ling H. Resolvin D1 suppresses pannus formation via decreasing connective tissue growth factor caused by upregulation of miRNA-146a-5p in rheumatoid arthritis. Arthritis Res Ther. (2020) 22:61. 10.1186/s13075-020-2133-2 32216830 PMC7099804

[75] Benabdoun HA Kulbay M Rondon EP Vallières F Shi Q Fernandes J. *In vitro*and *in vivo* assessment of the proresolutive and antiresorptive actions of resolvin D1: relevance to arthritis. Arthritis Res Ther. (2019) 21:72. 10.1186/s13075-019-1852-8 30867044 PMC6416871

[76] Norling LV Headland SE Dalli J Arnardottir HH Haworth O Jones HR. Proresolving and cartilage-protective actions of resolvin D1 in inflammatory arthritis. JCI Insight. (2016) 1:e85922. 10.1172/jci.insight.85922 27158677 PMC4855303

[77] Flak MB Koenis DS Sobrino A Smith J Pistorius K Palmas F. GPR101 mediates the pro-resolving actions of RvD5n-3 DPA in arthritis and infections. J Clin Invest. (2020) 130:359–73. 10.1172/JCI131609 31793912 PMC6934227

[78] Alivernini S MacDonald L Elmesmari A Finlay S Tolusso B Gigante MR. Distinct synovial tissue macrophage subsets regulate inflammation and remission in rheumatoid arthritis. Nat Med. (2020) 26:1295–306. 10.1038/s41591-020-0939-8 32601335

[79] Huang CC Tseng TT Liu SC Lin YY Law YY Hu SL. S1P Increases VEGF production in osteoblasts and facilitates endothelial progenitor cell angiogenesis by inhibiting miR-16–5p expression via the c-Src/FAK signaling pathway in rheumatoid arthritis. Cells. (2021) 10:2168. 10.3390/cells10082168 34440937 PMC8393529

[80] Ichinose Y Eguchi K Migita K Kawabe Y Tsukada T Koji T. Apoptosis induction in synovial fibroblasts by ceramide: *in vitro* and *in vivo* effects. J Lab Clin Med. (1998) 131:410–6. 10.1016/S0022-2143(98)90141-X 9605105

[81] Migita K Honda S Yamasaki S Hirai Y Fukuda T Aoyagi T. Regulation of rheumatoid synovial cell growth by ceramide. Biochem Biophys Res Commun. (2000) 269:70–5. 10.1006/bbrc.2000.2239 10694479

[82] Inoue T Kohno M Nagahara H Murakami K Sagawa T Kasahara A. Upregulation of sphingosine-1-phosphate receptor 3 on fibroblast-like synoviocytes is associated with the development of collagen-induced arthritis via increased interleukin-6 production. PLoS ONE. (2019) 14:e0218090. 10.1371/journal.pone.0218090 31173610 PMC6555509

[83] Sun M Deng R Wang Y Wu H Zhang Z Bu Y. Sphingosine kinase 1/sphingosine 1-phosphate/sphingosine 1-phosphate receptor 1 pathway: a novel target of geniposide to inhibit angiogenesis. Life Sci. (2020) 256:117988. 10.1016/j.lfs.2020.117988 32569777

[84] Arias de la Rosa I Escudero-Contreras A Rodríguez-Cuenca S Ruiz-Ponce M Jiménez-Gómez Y Ruiz-Limón P. Defective glucose and lipid metabolism in rheumatoid arthritis is determined by chronic inflammation in metabolic tissues. J Intern Med. (2018) 284:61–77. 10.1111/joim.12743 29532531

[85] Toms TE Panoulas VF Smith JP Douglas KM Metsios GS Stavropoulos-Kalinoglou A. Rheumatoid arthritis susceptibility genes associate with lipid levels in patients with rheumatoid arthritis. Ann Rheum Dis. (2011) 70:1025–32. 10.1136/ard.2010.144634 21398331

[86] Hu HJ Jin EH Yim SH Yang SY Jung SH Shin SH. Common variants at the promoter region of the APOM confer a risk of rheumatoid arthritis. Exp Mol Med. (2011) 43:613–21. 10.3858/emm.2011.43.11.068 21844665 PMC3249587

[87] Julià A Ballina J Cañete JD Balsa A Tornero-Molina J Naranjo A. Genome-wide association study of rheumatoid arthritis in the Spanish population: KLF12 as a risk locus for rheumatoid arthritis susceptibility. Arthritis Rheum. (2008) 58:2275–86. 10.1002/art.23623 18668548

[88] Freudenberg J Lee HS Han BG Shin HD Kang YM Sung YK. Genome-wide association study of rheumatoid arthritis in Koreans: population-specific loci as well as overlap with European susceptibility loci. Arthritis Rheum. (2011) 63:884–93. 10.1002/art.30235 21452313

[89] Terao C Yamada R Ohmura K Takahashi M Kawaguchi T Kochi Y. The human AIRE gene at chromosome 21q22 is a genetic determinant for the predisposition to rheumatoid arthritis in Japanese population. Hum Mol Genet. (2011) 20:2680–5. 10.1093/hmg/ddr161 21505073

[90] Myouzen K Kochi Y Okada Y Terao C Suzuki A Ikari K. Functional variants in NFKBIE and RTKN2 involved in activation of the NF-κB pathway are associated with rheumatoid arthritis in Japanese. PLoS Genet. (2012) 8:e1002949. 10.1371/journal.pgen.1002949 23028356 PMC3441678

[91] Tokuhiro S Yamada R Chang X Suzuki A Kochi Y Sawada T. An intronic SNP in a RUNX1 binding site of SLC22A4, encoding an organic cation transporter, is associated with rheumatoid arthritis. Nat Genet. (2003) 35:341–8. 10.1038/ng1267 14608356

[92] Newman B Wintle RF van Oene M Yazdanpanah M Owen J Johnson B. SLC22A4 polymorphisms implicated in rheumatoid arthritis and Crohn's disease are not associated with rheumatoid arthritis in a Canadian Caucasian population. Arthritis Rheum. (2005) 52:425–9. 10.1002/art.20854 15693005

[93] Gieger C Geistlinger L Altmaier E Hrabé de Angelis M Kronenberg F Meitinger T. Genetics meets metabolomics: a genome-wide association study of metabolite profiles in human serum. PLoS Genet. (2008) 4:e1000282. 10.1371/journal.pgen.1000282 19043545 PMC2581785

[94] Consortium WTCC. Genome-wide association study of 14,000 cases of seven common diseases and 3,000 shared controls. Nature. (2007) 447:661–78. 10.1038/nature05911 17554300 PMC2719288

[95] Demirkan A van Duijn CM Ugocsai P Isaacs A Pramstaller PP Liebisch G. Genome-wide association study identifies novel loci associated with circulating phospho- and sphingolipid concentrations. PLoS Genet. (2012) 8:e1002490. 10.1371/journal.pgen.1002490 22359512 PMC3280968

[96] Massey J Plant D Hyrich K Morgan AW Wilson AG Spiliopoulou A. Genome-wide association study of response to tumour necrosis factor inhibitor therapy in rheumatoid arthritis. Pharmacogenomics J. (2018) 18:657–64. 10.1038/s41397-018-0040-6 30166627 PMC6150911

[97] Coras R Pedersen B Narasimhan R Brandy A Mateo L Prior-Español A. Imbalance between omega-6- and omega-3-derived bioactive lipids in arthritis in older adults. J Gerontol A Biol Sci Med Sci. (2021) 76:415–25. 10.1093/gerona/glaa113 32361743 PMC7907486

[98] Rodríguez-Carrio J Coras R Alperi-López M López P Ulloa C Ballina-García FJ. Profiling of serum oxylipins during the earliest stages of rheumatoid arthritis. Arthritis Rheumatol. (2021) 73:401–13. 10.1002/art.41537 33001576 PMC7914204

[99] Özgül Özdemir RB Soysal Gündüz Ö Özdemir AT Akgül Ö. Low levels of pro-resolving lipid mediators lipoxin-A4, resolvin-D1 and resolvin-E1 in patients with rheumatoid arthritis. Immunol Lett. (2020) 227:34–40. 10.1016/j.imlet.2020.08.006 32818598

[100] Beyer K Lie SA Bjørndal B Berge RK Svardal A Brun JG. Lipid, fatty acid, carnitine- and choline derivative profiles in rheumatoid arthritis outpatients with different degrees of periodontal inflammation. Sci Rep. (2021) 11:5332. 10.1038/s41598-021-84122-y 33674638 PMC7935865

[101] Luan H Gu W Li H Wang Z Lu L Ke M. Serum metabolomic and lipidomic profiling identifies diagnostic biomarkers for seropositive and seronegative rheumatoid arthritis patients. J Transl Med. (2021) 19:500. 10.1186/s12967-021-03169-7 34876179 PMC8650414

[102] Gomez EA Colas RA Souza PR Hands R Lewis MJ Bessant C. Blood pro-resolving mediators are linked with synovial pathology and are predictive of DMARD responsiveness in rheumatoid arthritis. Nat Commun. (2020) 11:5420. 10.1038/s41467-020-19176-z 33110080 PMC7591509

[103] Surowiec I Ärlestig L Rantapää-Dahlqvist S Trygg J. Metabolite and lipid profiling of biobank plasma samples collected prior to onset of rheumatoid arthritis. PLoS ONE. (2016) 11:e0164196. 10.1371/journal.pone.0164196 27755546 PMC5068821

[104] Coras R Kavanaugh A Boyd T Huynh Q Pedersen B Armando AM. Pro- and anti-inflammatory eicosanoids in psoriatic arthritis. Metabolomics. (2019) 15:65. 10.1007/s11306-019-1527-0 31004236 PMC6533065

[105] Coras R Kavanaugh A Kluzniak A Holt D Weilgosz A Aaron A. Differences in oxylipin profile in psoriasis versus psoriatic arthritis. Arthritis Res Ther. (2021) 23:200. 10.1186/s13075-021-02575-y 34303373 PMC8310583

[106] Looby N Roszkowska A Reyes-Garcés N Yu M Baczek T Kulasingam V. Serum metabolic fingerprinting of psoriasis and psoriatic arthritis patients using solid-phase microextraction-liquid chromatography-high-resolution mass spectrometry. Metabolomics. (2021) 17:59. 10.1007/s11306-021-01805-3 34137950 PMC8211611

[107] Kosinska M Liebisch G Wilhelm J Ishaque B Rickert M Steinmeyer J. Lipidomic analysis of human serum reveals elevated phospho-and sphingolipid species levels during osteoarthritis. Osteoarthritis and Cartilage. (2018) 26:S169–70. 10.1016/j.joca.2018.02.368

[108] Van de Vyver A Clockaerts S van de Lest CHA Wei W Verhaar J Van Osch GJVM. Synovial fluid fatty acid profiles differ between osteoarthritis and healthy patients. Cartilage. (2020) 11:473–8. 10.1177/1947603518798891 30203669 PMC7488810

[109] Sewell GW Hannun YA Han X Koster G Bielawski J Goss V. Lipidomic profiling in Crohn's disease: abnormalities in phosphatidylinositols, with preservation of ceramide, phosphatidylcholine and phosphatidylserine composition. Int J Biochem Cell Biol. (2012) 44:1839–46. 10.1016/j.biocel.2012.06.016 22728312 PMC3778899

[110] Low PC Misaki R Schroder K Stanley AC Sweet MJ Teasdale RD. Phosphoinositide 3-kinase δ regulates membrane fission of Golgi carriers for selective cytokine secretion. J Cell Biol. (2010) 190:1053–65. 10.1083/jcb.201001028 20837769 PMC3101599

[111] Diab J Hansen T Goll R Stenlund H Ahnlund M Jensen E. Lipidomics in Ulcerative Colitis Reveal Alteration in Mucosal Lipid Composition Associated With the Disease State. Inflamm Bowel Dis. (2019) 25:1780–7. 10.1093/ibd/izz098 31077307

[112] Elvas F Stroobants S Wyffels L. Phosphatidylethanolamine targeting for cell death imaging in early treatment response evaluation and disease diagnosis. Apoptosis. (2017) 22:971–87. 10.1007/s10495-017-1384-0 28623512

[113] Delvaeye T Wyffels L Deleye S Lemeire K Gonçalves A Decrock E. Noninvasive whole-body imaging of phosphatidylethanolamine as a cell death marker using. J Nucl Med. (2018) 59:1140–5. 10.2967/jnumed.117.205815 29419481

[114] Pearl DS Masoodi M Eiden M Brümmer J Gullick D McKeever TM. Altered colonic mucosal availability of n-3 and n-6 polyunsaturated fatty acids in ulcerative colitis and the relationship to disease activity. J Crohns Colitis. (2014) 8:70–9. 10.1016/j.crohns.2013.03.013 23619007

[115] Masoodi M Pearl DS Eiden M Shute JK Brown JF Calder PC. Altered colonic mucosal Polyunsaturated Fatty Acid (PUFA) derived lipid mediators in ulcerative colitis: new insight into relationship with disease activity and pathophysiology. PLoS ONE. (2013) 8:e76532. 10.1371/journal.pone.0076532 24204637 PMC3799829

[116] Diab J Al-Mahdi R Gouveia-Figueira S Hansen T Jensen E Goll R. A quantitative analysis of colonic mucosal oxylipins and endocannabinoids in treatment-naïve and deep remission ulcerative colitis patients and the potential link with cytokine gene expression. Inflamm Bowel Dis. (2019) 25:490–7. 10.1093/ibd/izy349 30476077 PMC6383859

[117] Bazarganipour S Hausmann J Oertel S El-Hindi K Brachtendorf S Blumenstein I. The lipid status in patients with ulcerative colitis: sphingolipids are disease-dependent regulated. J Clin Med. (2019) 8:971. 10.3390/jcm8070971 31277430 PMC6678307

[118] Li Z Kabir I Tietelman G Huan C Fan J Worgall T. Sphingolipid de novo biosynthesis is essential for intestine cell survival and barrier function. Cell Death Dis. (2018) 9:173. 10.1038/s41419-017-0214-1 29415989 PMC5833386

[119] Nakayama H Ogawa H Takamori K Iwabuchi K. GSL-enriched membrane microdomains in innate immune responses. Arch Immunol Ther Exp. (2013) 61:217–28. 10.1007/s00005-013-0221-6 23456206

[120] Sato T Iwabuchi K Nagaoka I Adachi Y Ohno N Tamura H. Induction of human neutrophil chemotaxis by *Candida albicans*-derived beta-1,6-long glycoside side-chain-branched beta-glucan. J Leukoc Biol. (2006) 80:204–11. 10.1189/jlb.0106069 16670126

[121] Borodzicz S Rudnicka L Mirowska-Guzel D Cudnoch-Jedrzejewska A. The role of epidermal sphingolipids in dermatologic diseases. Lipids Health Dis. (2016) 15:13. 10.1186/s12944-016-0178-7 26786937 PMC4717587

[122] Lew BL Cho Y Kim J Sim WY Kim NI. Ceramides and cell signaling molecules in psoriatic epidermis: reduced levels of ceramides, PKC-alpha, and JNK. J Korean Med Sci. (2006) 21:95–9. 10.3346/jkms.2006.21.1.95 16479073 PMC2733987

[123] Cho Y Lew BL Seong K Kim NI. An inverse relationship between ceramide synthesis and clinical severity in patients with psoriasis. J Korean Med Sci. (2004) 19:859–63. 10.3346/jkms.2004.19.6.859 15608398 PMC2816304

[124] Checa A Xu N Sar DG Haeggström JZ Ståhle M Wheelock CE. Circulating levels of sphingosine-1-phosphate are elevated in severe, but not mild psoriasis and are unresponsive to anti-TNF-α treatment. Sci Rep. (2015) 5:12017. 10.1038/srep12017 26174087 PMC4502512

[125] Ilves L Ottas A Kaldvee B Abram K Soomets U Zilmer M. Metabolomic analysis of skin biopsies from patients with atopic dermatitis reveals hallmarks of inflammation, disrupted barrier function and oxidative stress. Acta Derm Venereol. (2021) 101:adv00407. 10.2340/00015555-3766 33585945 PMC9366688

[126] Sorokin AV Domenichiello AF Dey AK Yuan ZX Goyal A Rose SM. Bioactive lipid mediator profiles in human psoriasis skin and blood. J Invest Dermatol. (2018) 138:1518–28. 10.1016/j.jid.2018.02.003 29454560 PMC6121727

[127] Miller CC Ziboh VA. Induction of epidermal hyperproliferation by topical n-3 polyunsaturated fatty acids on guinea pig skin linked to decreased levels of 13-hydroxyoctadecadienoic acid (13-hode). J Invest Dermatol. (1990) 94:353–8. 10.1111/1523-1747.ep12874482 2106562

[128] Ogawa E Owada Y Ikawa S Adachi Y Egawa T Nemoto K. Epidermal FABP (FABP5) regulates keratinocyte differentiation by 13(S)-HODE-mediated activation of the NF-κB signaling pathway. J Invest Dermatol. (2011) 131:604–12. 10.1038/jid.2010.342 21068754

[129] Hattori T Obinata H Ogawa A Kishi M Tatei K Ishikawa O. G2A plays proinflammatory roles in human keratinocytes under oxidative stress as a receptor for 9-hydroxyoctadecadienoic acid. J Invest Dermatol. (2008) 128:1123–33. 10.1038/sj.jid.5701172 18034171

[130] Pipper C Bordag N Reiter B Economides K Florian P Birngruber T. LC/MS/MS analyses of open-flow microperfusion samples quantify eicosanoids in a rat model of skin inflammation. J Lipid Res. (2019) 60:758–66. 10.1194/jlr.M087221 30696699 PMC6446707

[131] Spector AA Fang X Snyder GD Weintraub NL. Epoxyeicosatrienoic acids (EETs): metabolism and biochemical function. Prog Lipid Res. (2004) 43:55–90. 10.1016/S0163-7827(03)00049-3 14636671

[132] Grant GE Rokach J Powell WS. 5-Oxo-ETE and the OXE receptor. Prostaglandins Other Lipid Mediat. (2009) 89:98–104. 10.1016/j.prostaglandins.2009.05.002 19450703 PMC2906239

[133] Shao S Chen J Swindell WR Tsoi LC Xing X Ma F. Phospholipase A2 enzymes represent a shared pathogenic pathway in psoriasis and pityriasis rubra pilaris. JCI Insight. (2021) 6:e151911. 10.1172/jci.insight.151911 34491907 PMC8564909

[134] Fogh K Herlin T Kragballe K. Eicosanoids in acute and chronic psoriatic lesions: leukotriene B4, but not 12-hydroxy-eicosatetraenoic acid, is present in biologically active amounts in acute guttate lesions. J Invest Dermatol. (1989) 92:837–41. 10.1111/1523-1747.ep12696858 2542417

[135] Heitmann J Iversen L Kragballe K Ziboh VA. Incorporation of 15-hydroxyeicosatrienoic acid in specific phospholipids of cultured human keratinocytes and psoriatic plaques. Exp Dermatol. (1995) 4:74–8. 10.1111/j.1600-0625.1995.tb00225.x 7640878

[136] Xu J Duan X Hu F Poorun D Liu X Wang X. Resolvin D1 attenuates imiquimod-induced mice psoriasiform dermatitis through MAPKs and NF-κB pathways. J Dermatol Sci. (2018) 89:127–35. 10.1016/j.jdermsci.2017.10.016 29137840

[137] Liu GJ Tao T Zhang XS Lu Y Wu LY Gao YY. Resolvin D1 Attenuates innate immune reactions in experimental subarachnoid hemorrhage rat model. Mol Neurobiol. (2021) 58:1963–77. 10.1007/s12035-020-02237-1 33411245

[138] Zhang F Wei K Slowikowski K Fonseka CY Rao DA Kelly S. Defining inflammatory cell states in rheumatoid arthritis joint synovial tissues by integrating single-cell transcriptomics and mass cytometry. Nat Immunol. (2019) 20:928–42. 10.1038/s41590-019-0378-1 31061532 PMC6602051

